# Honey bee retinue workers respond similarly to queens despite seasonal differences in Queen Mandibular Pheromone (QMP) signaling

**DOI:** 10.1371/journal.pone.0291710

**Published:** 2023-09-28

**Authors:** Mark J. Carroll, Nicholas J. Brown, Zachary Ruetz, Vincent A. Ricigliano, Kirk E. Anderson

**Affiliations:** 1 Carl Hayden Bee Research Center USDA-ARS, Tucson, Arizona, United States of America; 2 Honey Bee Breeding, Genetics, and Physiology Research USDA-ARS, Baton Rouge, Louisiana, United States of America; 3 Department of Entomology and Center for Insect Science, University of Arizona, Tucson, Arizona, United States of America; University of Alberta, CANADA

## Abstract

Honey bee colonies maintain viable queens in part through communication with Queen Mandibular Pheromone (QMP), a mixture that signals the queen’s presence and reproductive quality to workers. In turn, workers are thought to provide retinue queen care or replace queens partially based on QMP profiles. We examined the effects of seasonal dearth (overwintering in a warm subtropical location) on queen-worker interactions. Retinue worker responses to continuously ovipositing queens were considered in view of QMP signaling and queen reproductive quality. QMP signaling was estimated from QMP residues recovered from nest worker bodies, which is the primary mode of QMP transfer from the queen to the colony at large. QMP residues varied seasonally but not at all with queen reproductive quality (spermatheca sperm storage, ovary protein and lipid contents). 9-HDA and 9-ODA were lower in January than other months. HOB decreased from July to January, while HVA, a component associated with mated queens, increased sharply in January. Despite these seasonal signaling differences, retinue workers attended queens at similar levels through the months. In terms of reproductive quality, queens did not differ over the months in matedness (spermatheca sperm storage) or physiological age (protein carbonyl content), but varied in nutrient allocation to reproductive and non-reproductive tissues. Queen ovaries contained more protein in September than in November, and more lipid in July and September than in November and January. Queen fat bodies had more protein in July than September or November, but less lipid in July and September than November or January. Retinue worker responses did not vary with seasonal QMP changes, but reflected overall continuous brood rearing efforts and queen matedness throughout the year. The absence of seasonal differences in worker responses to QMP should be considered in the broader context of continuous reproductive efforts in warm subtropical colonies.

## Introduction

A central challenge to honey bees (*Apis mellifera* L.) is the development and maintenance of colonies on unpredictable food resources under periods of variable stress. Colonies incur significant losses due to colony stressors such as poor nutrition, poor forage, environmental extremes, high pathogen infections and parasite infestations, queen failures, or a combination of these factors [1–7]. As a social unit, honey bees rely on pheromones and other communication systems to coordinate essential functions under highly changing conditions [8–13 for reviews and overviews]. However, less is known about how these communication and coordination systems function in times of colony stress when most losses occur. These colony stressors may affect not only pheromone production but also the responses of pheromone receivers and the context of colony needs.

One critical coordinated function of a honey bee colony is the maintenance of a well-mated queen, the only egg-laying colony member capable of producing female workers [11]. Queen losses and premature supersedures are broadly considered to be a major source of colony losses among beekeepers [7,14,15]. Colonies have historically retained queens for 3–5 years, but have experienced greater rates of queen supersedure, poor sperm storage, and queen failure in recent years [16–20]. Worker bees interact most directly with the queen through the retinue, a semicircular gathering of nest workers (average size 17.2 ± 0.3 SE workers (n = 93), Seeley, 1979 [21]) that provide food and essential care to the queen [21,22]. In the process of antennating, licking, feeding and grooming the queen, retinue workers disperse certain queen contact pheromones throughout the colony workers [23,24]. Key among the pheromone signals is Queen Mandibular Pheromone (QMP), a complex of five major components (methyl-*p-*hydroxybenzoate (HOB), 4-hydroxy-3-methoxyphenylethanol (HVA), 9-oxo-2-decenoic acid (9-ODA), and two enantiomers of 9-hydroxy-2-decenoic acid (9-HDA)) [25,26]. Along with 4 other pheromone compounds (coniferyl alcohol, 1-hexadecanol, linolenic acid, and methyl oleate), QMP compounds also function as Queen Retinue Pheromone (QRP), a pheromone complex that promotes retinue behaviors toward the queen [27]. These contact pheromones continuously spread across the body of the queen where they are removed by grooming and transferred by attending retinue workers bees to other workers in the colony [23,24]. QMP components are among the key pheromones thought to project the presence and quality of the queen to the colony at large [see 9 for review]. QMP promotes queenright characteristics among workers by suppressing worker ovarian development, suppressing the rearing of new queens, inhibiting worker production of queen-like pheromones, delaying worker maturation from nest bees to foragers, and calming queenless workers [28–33]. Together with other queen pheromones such as tergal glands and Dufour’s gland secretions, QMP modulates worker responses to queens [27,34–37]. In turn, workers may interact differentially with queens or queen extracts based on perceived reproductive quality (stored sperm amount, viability, and insemination volume), caste development, and physiological quality (injury, disease, and senescence) [35,38–50]. Queen pheromone signaling has been proposed to function as an underlying “honest signal” of queen quality that allows workers to detect and replace substandard queens. Alternatively, queen pheromone signaling has also been proposed as a method to enact “queen control” and suppression of worker reproduction. [9,28,42,46,51–53]. Workers are thought to respond to significant changes or declines in QMP by reducing queen care and initiating queen supersedure through rearing of replacement daughter queens [9,11,51]. However, researchers have had mixed results in demonstrating worker preferences for nominally high reproductive quality queens over lower quality queens in bioassays [see 36,47].

Less clear is how workers maintain healthy egg-laying queens during periods when the queen’s reproductive potential is not directly apparent. Colonies routinely experience forage scarcity and seasonal changes that severely impact colony nutrition and brood rearing. Colonies in subtropical and temperate locations often face extended dearths, such as overwintering, where forage is scarce and brood rearing is reduced or ceases entirely [54–57]. During extended dearths, queen reproductive quality, queen oviposition rates, and brood rearing may decline substantially compared to more productive times [58,59]. Successful maintenance of a viable queen through dearths is critical to future colony expansion once conditions improve. However, stressed queens may have the suboptimal characteristics of failing queens under certain conditions. Further complicating maintenance of a viable queen through these periods are impacts of external stressors on individual queen quality. Individual queens may show reduced oviposition, worker acceptance, or queen retention due not only to sperm depletion and age senescence, but also poor colony nutrition, injury, disease, or pesticide exposures [59–63].

In this experiment, we examined whether QMP pheromone signaling and retinue worker responses to queens closely reflected differences attributable to queen reproductive quality (i.e. “honest signaling”), or were affected by seasonal stress [9,51]. We specifically evaluated queens originating from a warm subtropical location where queen oviposition and brood rearing continue year round despite restricted forage availability [59]. Honey bees in warm subtropical regions such as the southern United States (including southern Arizona, south Florida, south Texas, and southern California) experience warmer conditions and shorter winters but may readily deplete limited food resources and worker populations through excessive brood rearing and foraging activity [56]. Despite less harsh winter conditions, colonies in these locations still incur heavy losses primarily due to scarce and unpredictable food resources exacerbated by brief but powerful cold snaps [55,56]. Commercial honey bee colonies from the Imperial Valley of California experience a seven month extended cropless period between early summer and late winter pollination [59]. Colonies kept in this environment subsist on marginal forage from roadsides, irrigation canals, and managed natural areas supplemented by pollen substitutes, yet rarely completely cease queen oviposition or brood rearing even in mid-winter. Previous studies on queen overwintering have largely been conducted in cooler subtropical or temperate locations where queen oviposition and brood rearing cease entirely for a longer period [54,57,58].

As part of a longitudinal study, we transferred queens from Imperial Valley, CA, USA colonies to colonies in Tucson, AZ, USA at four time points (July 2016, September 2016, November 2016, and January 2017) to assess queen quality, QMP signaling, and queen-worker interactions with unrelated workers in surrogate colonies [20]. Researchers have commonly used extracts or synthetic blends to assess pheromones responses of surrogate workers in isolation from other factors [25,27,34,35,64]. Here, however, we evaluated surrogate worker responses to whole live queens *in situ* to include other queen semiochemicals such as tergal gland and Dufour’s gland secretions as well as less characterized cues, and to better assess queen-worker interaction behaviors with standardized colony constructs [34,35,37,64–66]. We measured QMP in the form that most nest workers encounter these pheromones. Critically, most nest workers encounter QMP not from direct contact with the queen but rather through exposure to QMP residues present on other nest worker bodies [23,24]. These residues are derived overwhelmingly from QMP produced by the queen along with very minor amounts of QMP queen and queen-like pheromones produced by the workers themselves [9,52,67]. Both types of residues would likely be shared between queens, retinue workers and outlying workers by grooming and contact transfers between individuals [23,24]. To this end, we used a retinue enclosure method to sample messenger and retinue workers that removed and transferred QMP from the queen over a set period of time [56]. We characterized key worker responses such as retinue size and the incidence of worker feedings of queens. Trophallactic feeding of the queen by retinue workers serves as the primary nutrient conduit for queen productivity [11,21]. Likewise, we measured queen reproductive quality (spermatheca sperm storage and ovary nutrient allocations) rather than colony metrics of queen productivity (brood populations) given that colony workers frequently cull eggs and young larvae through brood cannibalism in stressful times [68]. Workers are believed to detect and replace sperm-depleted queens (“failing queens” or “drone layers”) that exhaust their stored sperm and can no longer produce fertilized (female) eggs, although this has not been fully demonstrated [11,36]. To determine whether queen physiological age reflects chronological age, we assessed accumulated oxidative damage of fat body proteins [69,70]. By examining changes in both pheromone signaling and receiver responses, we determined how this essential interaction functions under a routinely encountered colony stress.

## Materials and methods

### Experimental queens and commercial honey bee colonies

We compared QMP pheromone signaling, queen-retinue worker interactions, and queen reproductive and physiological quality in Italian queens that originated from commercial colonies (Ashurst Bee Co., Inc) maintained in the Imperial Valley of California, USA from July 2016 to January 2017. These source colonies were previously used to pollinate almonds (mid-February to early March 2016) and seed alfalfa (mid-March to early July 2016) before entering a seven month cropless period (early July to mid-February) until the next almond pollination [59]. During this partial dearth, source colonies were placed in one of 17 apiaries in the Imperial Valley with limited forage. Colonies were given supplemental feed (12 L sucrose syrup and 2 kg pollen substitute) to supplement forage from roadside margins, irrigation canals, and natural areas. Despite marginal forage availability, almost all source colonies maintained pollen stores and continuously reared brood through the cropless period. Queens selected for the experiment were maximally from 16 to 23 months old during the experiment (time since last beekeeper requeening during post-almond colony splits). Any queens that replaced the original requeening queen were openly mated in apiaries densely populated by commercial colonies headed by queens of the same stock.

### Transfer and establishment of experimental queens

To standardize worker responses, we evaluated worker retinue retinue responses to queens in recently dequeened colonies of similar size and worker composition. Experimental queens were transferred from their original California source colonies to recently dequeened receiving surrogate colonies in Arizona for evaluation in July 2016, September 2016, November 2016, and January 2017. At each time point, between 20 to 45 queens were individually removed into queen cages and transported to an apiary at the University of Arizona Campus Agricultural Center (CAC, Tucson, Arizona, 32.27636, -110.93845, 719 m). Colonies at the Tucson site experienced major forage availability from late March to early June and minor forage periods from late July through October and mid-January through late March, with pronounced mid-summer (mid-June to late July) and early winter (November through early January) forage dearths [71]. Each receiving colony consisted of a 5 frame nucleus colony containing from 8,000 to 12,000 workers and 800 to 6,000 brood maintained in an apiary trailer at temperatures between 18°C to 35°C. Transferred queens were released from the queen cage after 24 h and monitored for oviposition and acceptance by the workers. Over 93% of the queens from Imperial Valley colonies were accepted by their receiving colonies over the course of the experiment.

### Queen pheromone transfers from the queen to retinue workers

We estimated QMP transfers from the queen to retinue workers by a recently developed *in situ* frame assay three days after queen acceptance [56]. We quantified the amount of QMP components present on the bodies of nest workers (including retinue workers) that were enclosed with the queen for a set amount of time. Approximately 60 to 80 workers were enclosed with the queen under a double screen metal mesh push-in cage embedded into the wax comb. This barrier allowed the enclosed workers to interact freely with the queen but prevented the caged workers from transferring QMP to outside workers. After two hours, bees outside the cage were gently brushed off and the frame briefly lowered into a large plastic container with a CO_2_ atmosphere to partially anaesthetize the enclosed bees. The queen was quickly removed from the push-in cage and carefully re-introduced to the surrogate colony periphery. All the remaining bees inside the cage were gased further until fully immobile and then collected on dry ice. No attempt was made to distinguish between workers that did and did not engage in retinue behaviors. The retinue worker sample was stored in the dark at -80°C until QMP extraction to minimize pheromone degradation.

QMP compounds were extracted from retinue workers, derivatized, and analyzed by GC-MS [26,56]. Sample workers were ground in liquid nitrogen and transferred to a glass Mason jar containing 30 mL diethyl ether and 100 μg cis-10-heptadecanoic acid (internal standard). The jar contents were then sealed, wrapped in aluminum foil, and extracted at 25°C for 20h in the dark. After extraction, 1.000 mL of the supernatant was transferred into a crimp vial, dried down, and silylated with 150 uL BSA (N,O-bis(trimethylsilyl)acetamide, Sigma, St. Louis, MO, USA) at 25°C for 16h in the dark. One hundred μL of the silylated solution was removed to a 200 μL vial glass insert and centrifuged to pellet particulates. Silylated compounds were separated in EI mode on a HP 7890A/5975D GC-MS. Exactly 1.0 μL sample was injected at 220°C onto an Agilent HP-5MS column (30 m x 0.250 mm ID x 0.25 μm film; Agilent Technologies, Santa Clara, CA, USA) at a flow rate of 1.2 mL/min. Compounds were separated by oven temperatures programmed from 40°C (0.5 min hold) to 220°C (15 min final hold) at 15°C/min. QMP compounds were identified by comparison of mass spectra and retention times with authentic standards (generously donated by Contech Inc., Vancouver, BC, Canada; Sigma, St. Louis, MO, USA). Five major QMP compounds (reported as four since the enatiomers of 9-HDA were indistiguishable) were detected on the bodies of retinue workers. Compound amounts were quantified by comparison of characteristic m/z fragment peak areas with synthetic standards in SIM mode. The fraction of the total sample analyzed was calculated from the amount of internal standard detected in the injected sample. Statistical comparisons were made of relative compound amounts normalized against the synthetic standards.

### Queen-retinue worker interactions

We assessed the relative attractiveness of individual queens recently introduced to unrelated workers by quantifying retinue worker numbers in observation colonies. Immediately after the sampling of QMP retinue workers, each receiving surrogate colony was converted from a standard 5 Langstroth frame nucleus colony to an observation colony. Three frames from the colony center were mounted vertically in a stacked configuration between acrylic sheet walls. Frame face-sized hinged doors were built into the acrylic sheet walls to allow easy access to the comb surfaces. Observation colonies were maintained either in the dark or under red light at an ambient room temperature of 30°C to 32°C to minimize disturbance of nest activities.

We recorded the formation and dissolution of queen retinues by colony workers over a 30 min period. Queen-worker interactions were videorecorded by a Sony HandiCam camcorder (mounted on a tripod with a 16 or 32 MB data card) with the video centered on the ever moving queen. The most numerous worker retinue count was noted for each one minute interval from the video recordings. Workers were rated as retinue workers if they were 1) touching the queen, 2) oriented toward the queen, or 3) stationary or nearly stationary for 10 seconds facing the queen (criteria after Seeley, 1979 [21]). Queens were compared based on the average size of their top three retinue formations. Intervals that did not have a formed retinue were recorded as such. The number of workers that successfully fed or attempted to feed the queen were also quantified. Worker attempts to feed the queen were characterized by 1) the worker remaining stationary facing the queen, 2) the extension of worker mouthparts toward the queen, and for successful feedings, 3) the extension of queen mouthparts to engage in trophallactic feeding for a few seconds to a few minutes (criteria after van der Blom, 1992 [22]). At the end of the observation period, the queen was sampled onto dry ice for later analysis of reproductive and physiological metrics.

### Queen mass and spermatheca sperm content

Frozen queens were first weighed to obtain whole body fresh mass. The fat body was removed intact while attached to abdominal tergites and sternites. Whole ovaries and the spermatheca were also removed for separate analyses. The number of sperm present in the queen spermatheca was estimated from hemocytometer counts [40]. The queen spermatheca was dissected into 950 μL HEPES pH 7.4 buffer and 50 μL 10% Bovine Serum Albumin (BSA; Sigma, St. Louis, MO, USA) and slightly vortexed. The sperm suspension was stained with 5 μL 2.4 mM propidium iodide (Live/Dead Sperm Viability kit, Thermo Fisher Scientific, Waltham, MA, USA) to detect dead (previously frozen) sperm. One μL stained suspension was visualized on a hemocytometer by fluorescence microscopy (Nikon Eclipse 80i microscope with D-FL Epi Fluorescence attachment, Nikon Instruments, Melville, NY, USA) at a fluorescence emission of 617 nm.

### Ovariole development and ovary nutrient contents

Queen ovaries were dissected into 2 mL 100 mM pH 7.4 HEPES buffer. The right ovary was first sectioned and carefully teased apart under a dissecting microscope to obtain counts of ovariole numbers [63]. Both ovaries were then combined and homogenized in the HEPES buffer for 30 sec by Bead Beater (BioSpec Products, Bartlesville, OK, USA). The ovary homogenate was analyzed for total soluble protein by a bicinchoninic acid (BCA) assay (Pierce BCA Protein Assay kit, Thermo Fisher Scientific, Waltham, MA, USA). Twenty five μL of the ovary homogenate was reacted with 175 μL BCA reactant solution in a 96 well plate at 32°C. Total soluble protein was estimated by comparing sample 562 nm absorbance against a bovine saline albumin (BSA; Sigma Inc., St. Louis, MO, USA) standard curve in a Gen-5 Plate Reader (Biotek, Inc., Winooski, UT, USA).

Total lipid content of the ovaries was quantified by a modified chromic acid assay on Folch extracts [72]. Exactly 510 μL ovary homogenate was combined with 1000 μL 2:1 chloroform: methanol, homogenized for 30 sec, and centrifuged to obtain a partitioned Folch extract. Eighty μL of the lower chloroform: methanol layer was transferred to a crimp seal vial, dried down, and reacted with 1.000 mL chromic acid at 95°C for 1h. Total lipid content was quantified by comparing the 620 nm absorbance of sample reactant solution against a reacted oleic acid (Sigma Inc., St. Louis, MO, USA) standard curve in a Gen-5 Plate Reader.

### Soluble protein and lipid contents of queen fat bodies

Fat body tissues were placed in 600 μL TE buffer, homogenized for 30 sec by Bead Beater, and centrifuged. Five μL of the fat body homogenate was reacted with 195 μL BCA reactant solution in a 96 well plate at 32°C and analyzed for total soluble protein content by BCA assay as previously described. Total lipid content of the queen fat bodies was quantified by performing a chromic acid oxidation assay on Folch extracts. One thousand μL chloroform and 210 μL DI water were added to a set fraction of the fat body homogenate, mixed thoroughly for 30 sec, and centrifuged to yield a bilayer partition. Then 80 μL of the lower chloroform: methanol layer was analyzed for total lipid content by a chromic acid oxidation assay as previously described.

### Physiological age (protein carbonyl content of fat bodies) of queens

The physiological age of each queen was estimated by quantifying accumulation of oxidized protein (protein carbonyl content) in fat body tissues (Protein Carbonyl Content Assay kit, Sigma, St. Louis, MO, USA)[69]. These oxidative stress products accumulate in tissues in a clock-like fashion and have previously been used to estimate the relative age of honey bees including queens ([69,70] but see Williams et al 2008 [73] for dissenting view, especially on use with metabolically active tissues). Four hundred μL fat body homogenate supernatant was incubated with streptozocin (10% final concentration) to precipitate nucleic acids. The resulting supernatant was reacted with 2,4-dinitrophenylhydrazine (DNPH), purified, and processed by the kit protocol. Purified protein pellets were resuspended in 100 μL 6M guanidine solution, transferred to a 96 well plate, and measured for 375 nm absorbance in a Gen-5 Plate Reader. For each queen, total protein carbonyl content was quantified by applying the protein carbonyl product (dinitrophenyl hydrazone adduct) millimolar extinction coefficient to the observed 375 nm absorbance reading. The protein carbonyl content was scaled against total soluble protein content as determined by a BCA assay.

### Statistical analysis

All statistical analyses except for queen feeding observations were performed with SAS 9.4 (SAS, Inc., Cary, NC, USA). The number of successful queen feedings and the number of successful and attempted queen feeding events were compared separately across time points by Pearson’s chi square test. Data sets were checked for normality by Shapiro-Wilks tests and examination of residuals (PROC UNIVARIATE). Ovariole number, retinue size, and queen wet mass were compared separately across time points by a one-way ANOVA (PROC GLM). Ovary protein content, fat body protein content, and fat body carbonyl content were log-transformed and compared by a one-way ANOVA. Data sets that lacked normality (QMP compound residues (HOB, HVA, 9-HDA, and 9-ODA), spermatheca sperm counts, ovary and fat body lipid contents) were compared by non-parametric Kruskal-Wallis tests at each time point. All significant tests were subjected to post hoc comparisons of treatment means (parametric) by Tukey’s HSD or treatment ranks (non-parametric) by Dwass, Steel, Critchlow-Fligner (DSCF) tests (PROC GLM; PROC NPAR1WAY dscf option). Principal component analysis was performed on the proportions of retinue worker QMP residue contents using PROC PRINCOMP. Initial variables were reduced to two principal components (PC1 and PC2) which were compared by non-parametric Kruskal-Wallis tests. Correlations were also made between individual QMP compound residue contents and each queen reproductive quality variable to determine the relationship between pheromone signaling and queen quality. To determine whether worker responses varied with QMP residues (HOB, HVA, 9-HDA, or 9-ODA) or queen reproductive quality (spermatheca sperm counts, ovary soluble protein, ovary lipid), we compared correlations between the dependent variable retinue size and each of these independent variables by Pearson correlation coefficients (parametric–ovary protein and retinues) or Spearman correlation coefficients (non-parametric–spermatheca sperm and ovary lipid)(PROC CORR with Bonferroni corrections).

## Results

### Queen-retinue worker interactions

**Fig 1:**
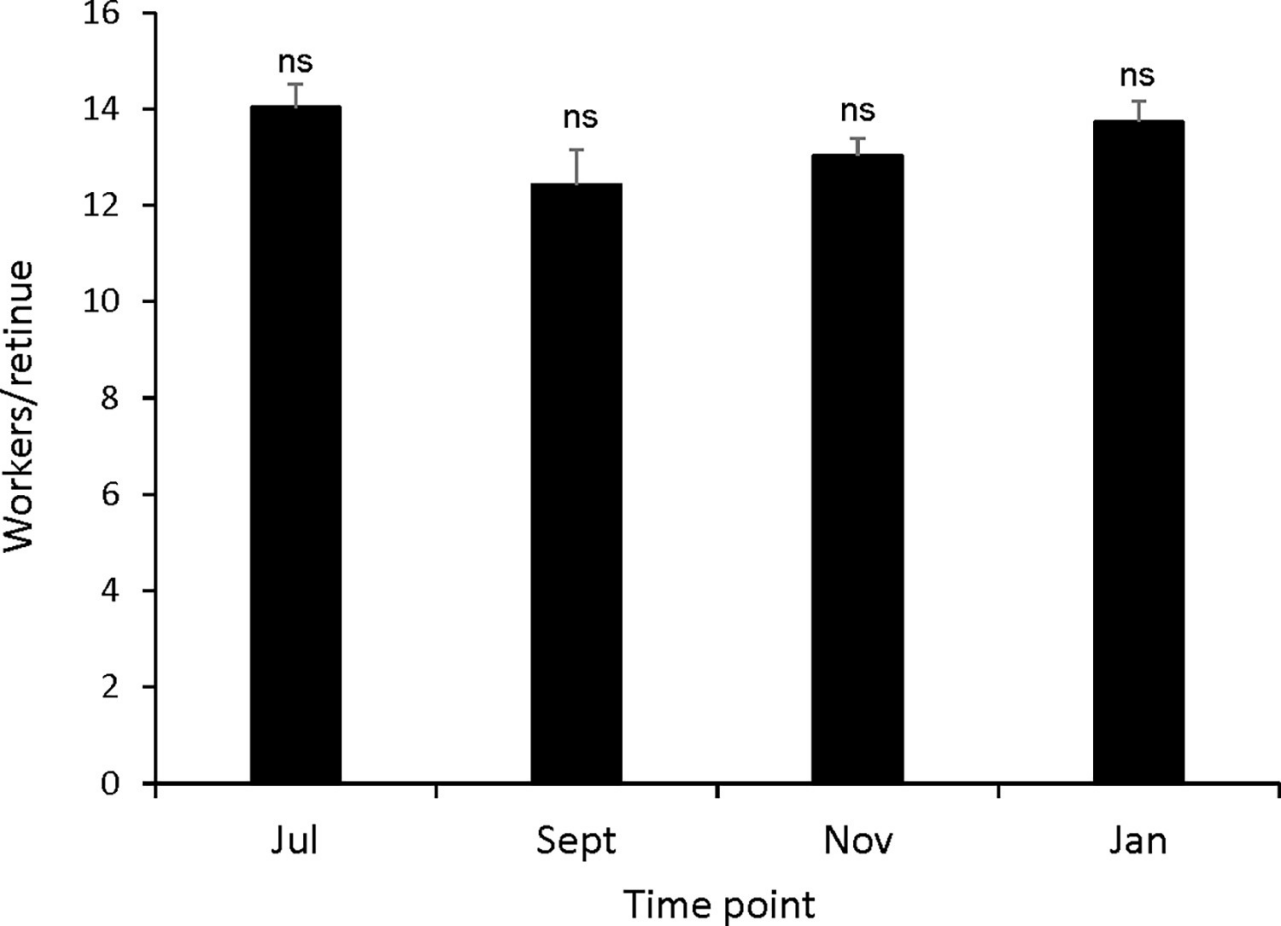
Number of retinue workers attending queens at different seasonal time points. Each number is the average value of the three largest retinues observed within 1 minute intervals over a 30 minute observation period. Error bars represent the standard error (N = 16 to 39 queens at each time point; none of the means differed by Tukey’s post hoc test (queen means; p>0.05)).

Queens were attended by similar sized worker retinues across all time points despite seasonal brood rearing differences in both source and receiving colonies (Fig 1, one-way ANOVA, F = 1.91, df = 3; p = 0.133). Workers also engaged in variable numbers of successful and attempted feedings with queens throughout the experiment despite lower colony brood production (S1 Fig, Pearson’s chi square test, Χ^2^ = 2.752, df = 3, p>0.05 (successful feedings only) and Χ^2^ = 12.960, df = 12, p>0.05 (both successful and attempted feedings)). The absence of seasonal differences in retinue sizes and feeding bouts is consistent with continuous queen oviposition activities observed in Imperial Valley and Tucson colonies throughout the experiment. Interestingly, numerous workers attempted to feed the queen (as indicated by worker position and extension of mouthparts) but she often rejected the majority of worker trophallactic feeding attempts.

### Queen pheromone transfers from the queen to retinue workers

**Fig 2:**
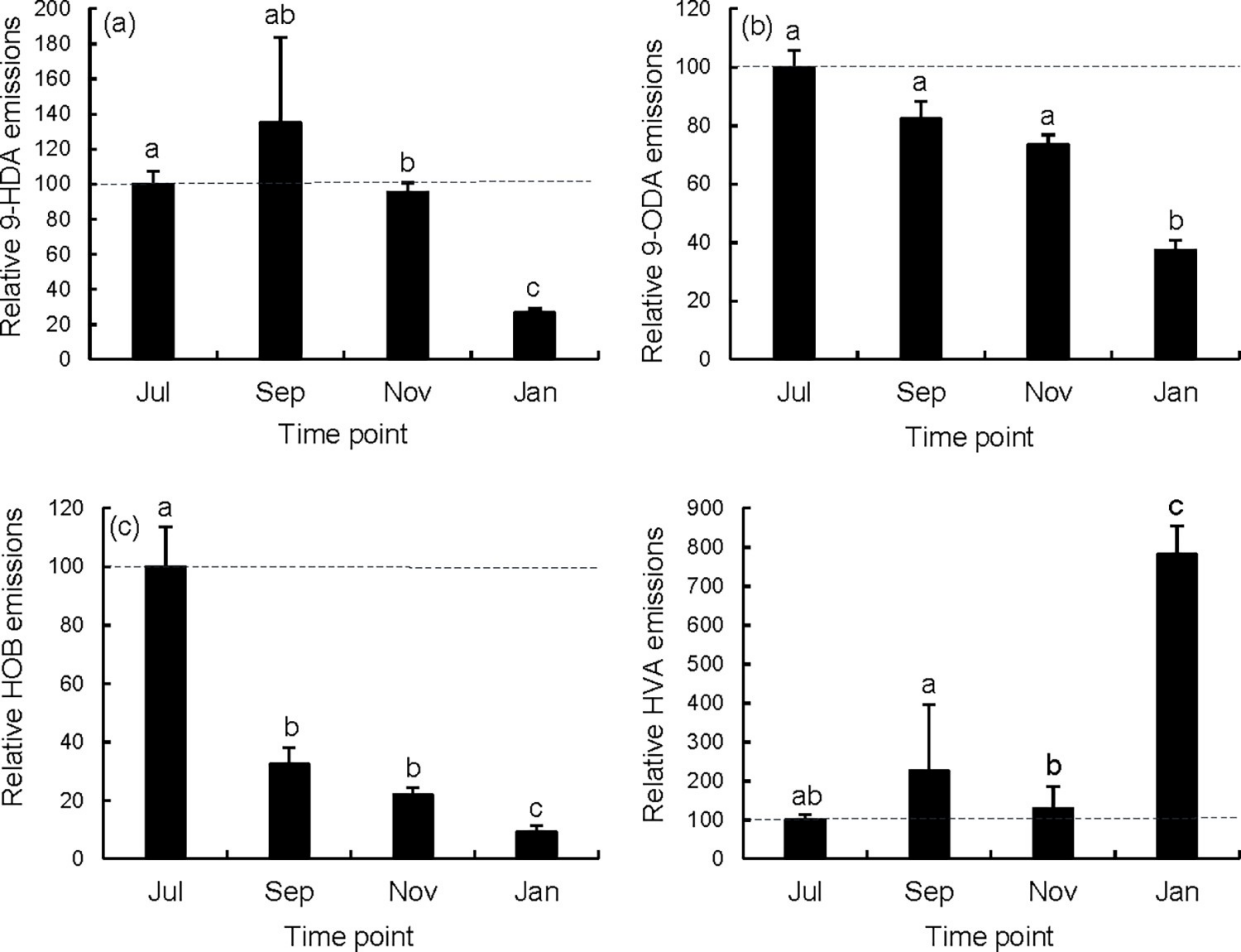
a-d. Relative proportions of QMP residues recovered from nest worker bodies at different seasonal time points. QMP components a) 9-hydroxy-2-decenoic acid (both enantiomers of 9-HDA), b) 9-oxo-2-decenoic acid (9-ODA), c) methyl-*p*-hydroxybenzoate (HOB), and d) 4-hydroxy-3-methoxyphenylethanol (HVA) were recovered from nest workers enclosed with the queen. Residue contents are shown relative to July residues. Error bars represent the standard error (N = 16 to 39 queens at each time point; means that do not share a superscript differ by DSCF multiple comparisons (queen ranks; p<0.05)).

QMP pheromone residues collected off nest worker bodies varied seasonally. The four QMP compound residue contents were reduced to two principal components PC1 and PC2 which explained 42.5% and 25.3% of the variance respectively (S2 Fig). Proportions of these four QMP residues differed significantly between treatment groups as indicated by comparisons across principal components (Kruskal-Wallis test, Χ^2^ = 49.445, df = 3, p<0.0001 (PC1); Χ^2^ = 37.098, df = 3, p<0.0001 (PC2)). Queen pheromone signaling (QMP component residues) from queens to retinue workers varied seasonally for all individual components (Fig 2A–2D, ranges for all four QMP components, Kruskal-Wallis test, ((HOB) Kruskal-Wallis test, Χ^2^ = 50.027, df = 3, p<0.0001; (HVA) Kruskal-Wallis test, Χ^2^ = 37.359, df = 3, p<0.0001; (9-HDA) Kruskal-Wallis test, Χ^2^ = 36.618, df = 3, p<0.0001; (9-ODA) Kruskal-Wallis test, Χ^2^ = 37.329, df = 3, p<0.0001)). 9-HDA and 9-ODA residues were significantly lower in January than July, September, or November. HOB residues steadily decreased from July to January. By contrast, HVA residues were sharply higher in January than other time points.

### Queen mass and spermatheca sperm content

Viable ovipositing queens were collected from all sampled colonies in the Imperial Valley across all time points. Almost all source and receiving colonies reared brood throughout the cropless period despite limited forage availability and the seasonal onset of subtropical winter. Experimental queens did not differ significantly in their fresh body mass by seasonal time point (S3 Fig, one-way ANOVA, F = 2.07, df = 3, p = 0.109). Queens showed similar levels of functional matedness (i.e. adequate amounts of sperm storage necessary for production of diploid female workers) across all time points (S4 Fig, Kruskal-Wallis test, Χ^2^ = 3.009, df = 3, p = 0.3903). Relatively few of the queens were well-mated by Woyke’s threshold of 3,000,000 sperm, but few of the queens sampled were significantly sperm-depleted (under 1,000,000 sperm) [74]. Of 102 queens examined, 32 had more than 3,000,000, 59 had between 1,000,000 and 3,000,000 sperm, 9 had between 1,000,000 and 100,000 sperm, and one had less than 100,000 sperm in her spermatheca.

### Ovariole development and ovary nutrient contents

**Fig 3:**
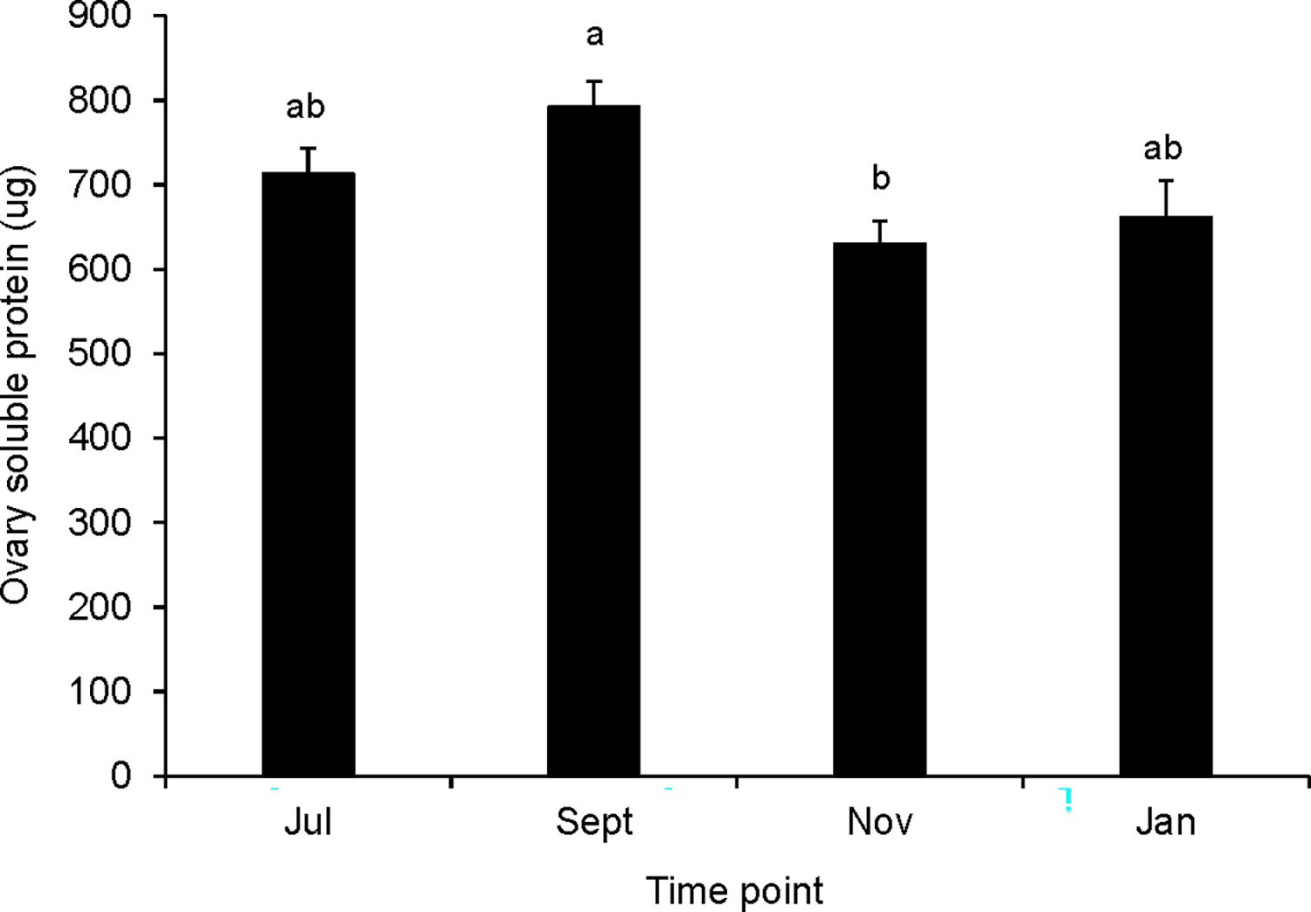
Total soluble protein contents of ovaries from queens sampled at different seasonal time points. Error bars represent the standard error (N = 15 to 39 queens at each time point; means that do not share a superscript differ by Tukey’s post hoc test (queen means; p<0.05)).

**Fig 4:**
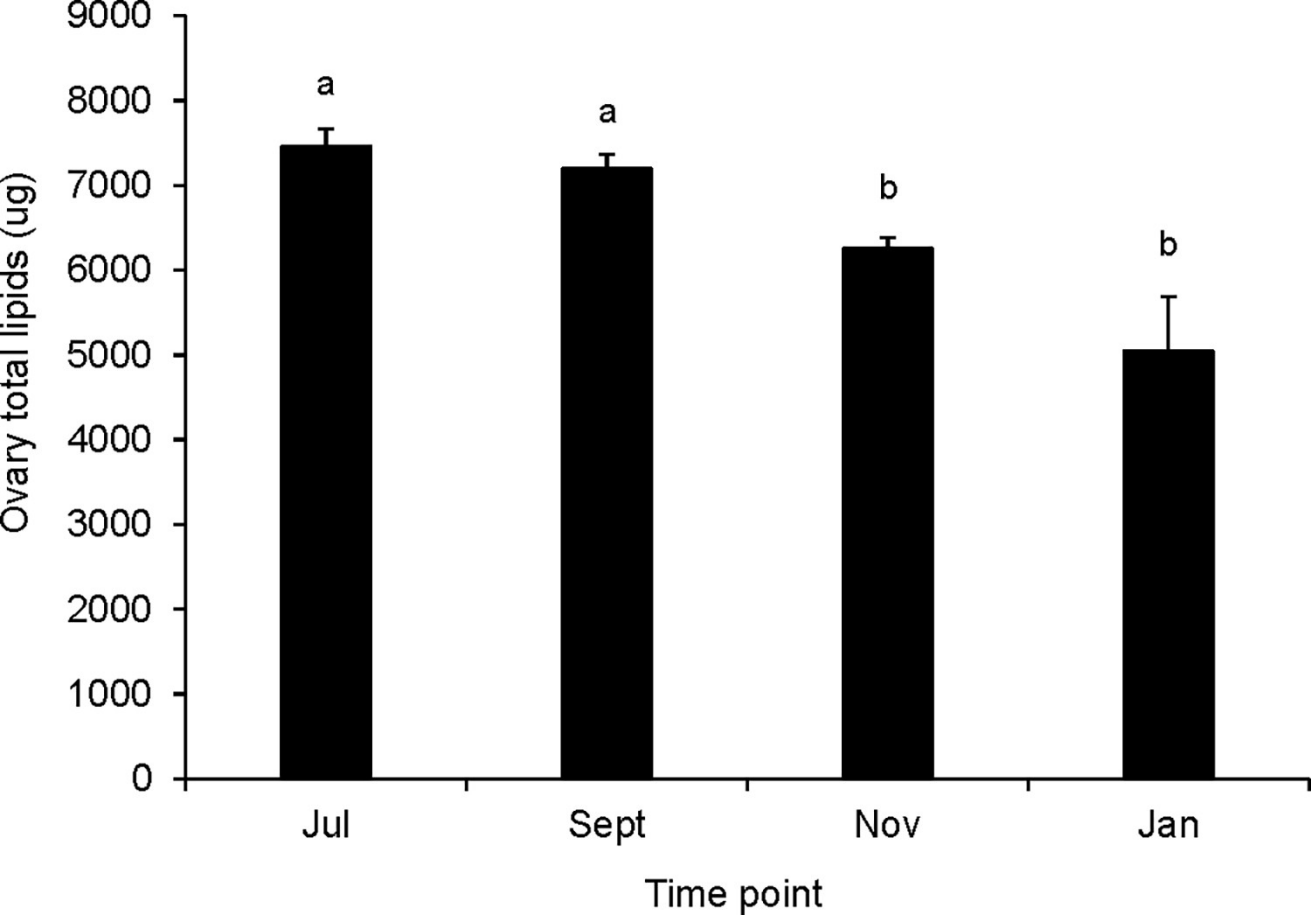
Total lipid contents of ovaries from queens sampled at different seasonal time points. Error bars represent the standard error (N = 15 to 39 queens at each time point; groups that do not share a superscript differ by DSCF multiple comparisons (queen ranks; p<0.05)).

Queens did not differ by time point in their ovary development as indicated by ovariole number, but varied in the amount of nutrients allocated to their ovaries throughout the study. Queens from different time points had similar numbers of ovarioles in their right ovaries (S5 Fig, one-way ANOVA, F = 1.31, df = 3, p = 0.277). However, ovary nutrient contents trended toward being lower in fall and winter queens than in summer queens. September queens had more soluble protein in their ovaries than queens sampled in November (Fig 3, one-way ANOVA, F = 5.92, df = 3, P<0.001). Queens sampled in July and September had more lipid in their ovaries than November and January queens (Fig 4, Kruskal-Wallis test, Χ^2^ = 31.486, df = 3, p<0.001). In general, these ovary nutrient allocation patterns reflected differences in brood production observed in Imperial Valley source colonies noted by our concurrent longitudinal study (i.e. declining brood production in fall and winter relative to mid and late summer) [59] as well as Tucson surrogate colonies.

### Soluble protein and lipid contents of queen fat bodies

**Fig 5:**
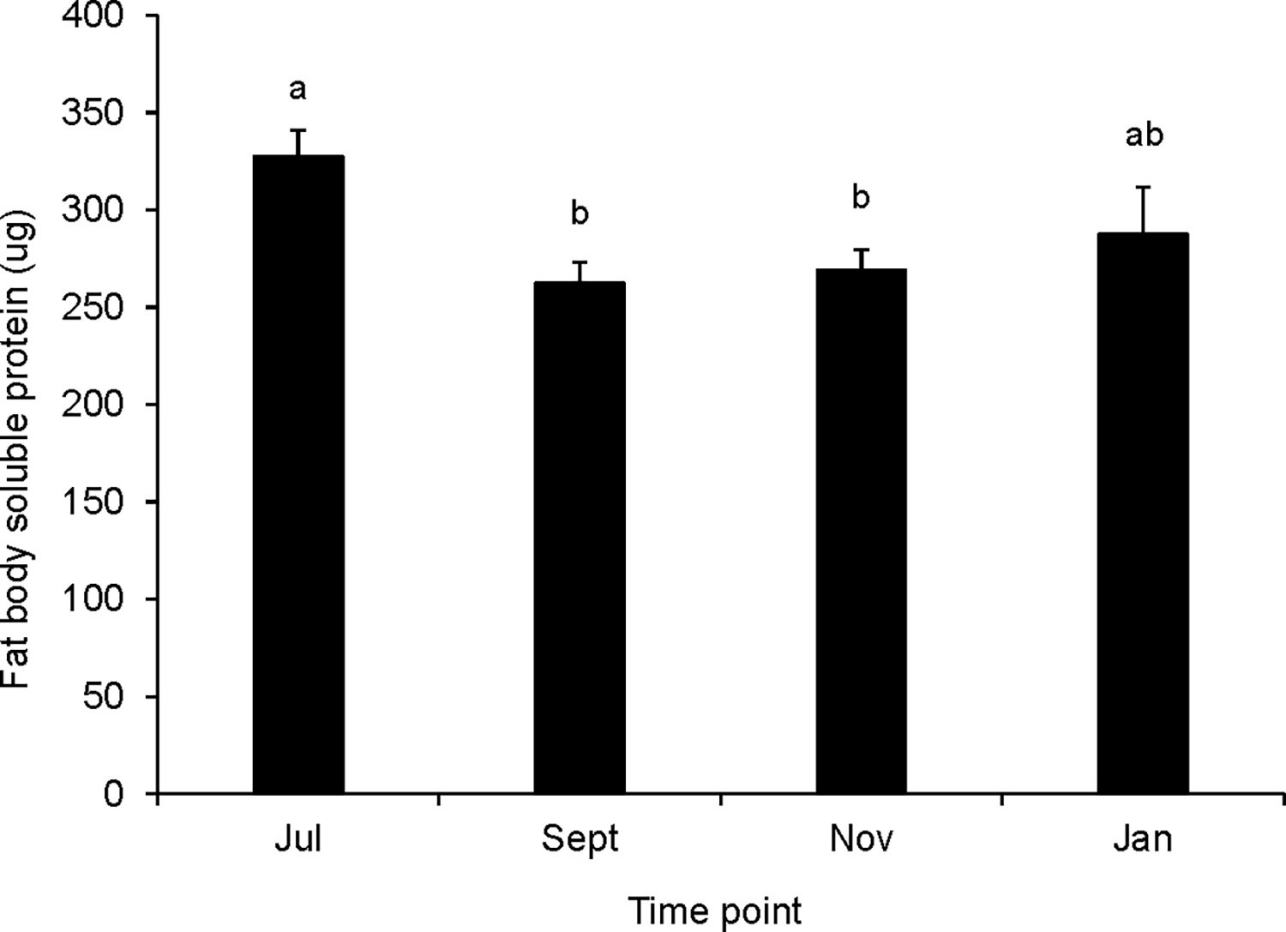
Total soluble protein contents of fat bodies from queens sampled at different seasonal time points. Error bars represent the standard error (N = 16 to 39 queens at each time point; means that do not share a superscript differ by Tukey’s post hoc test (queen means; p<0.05)).

**Fig 6:**
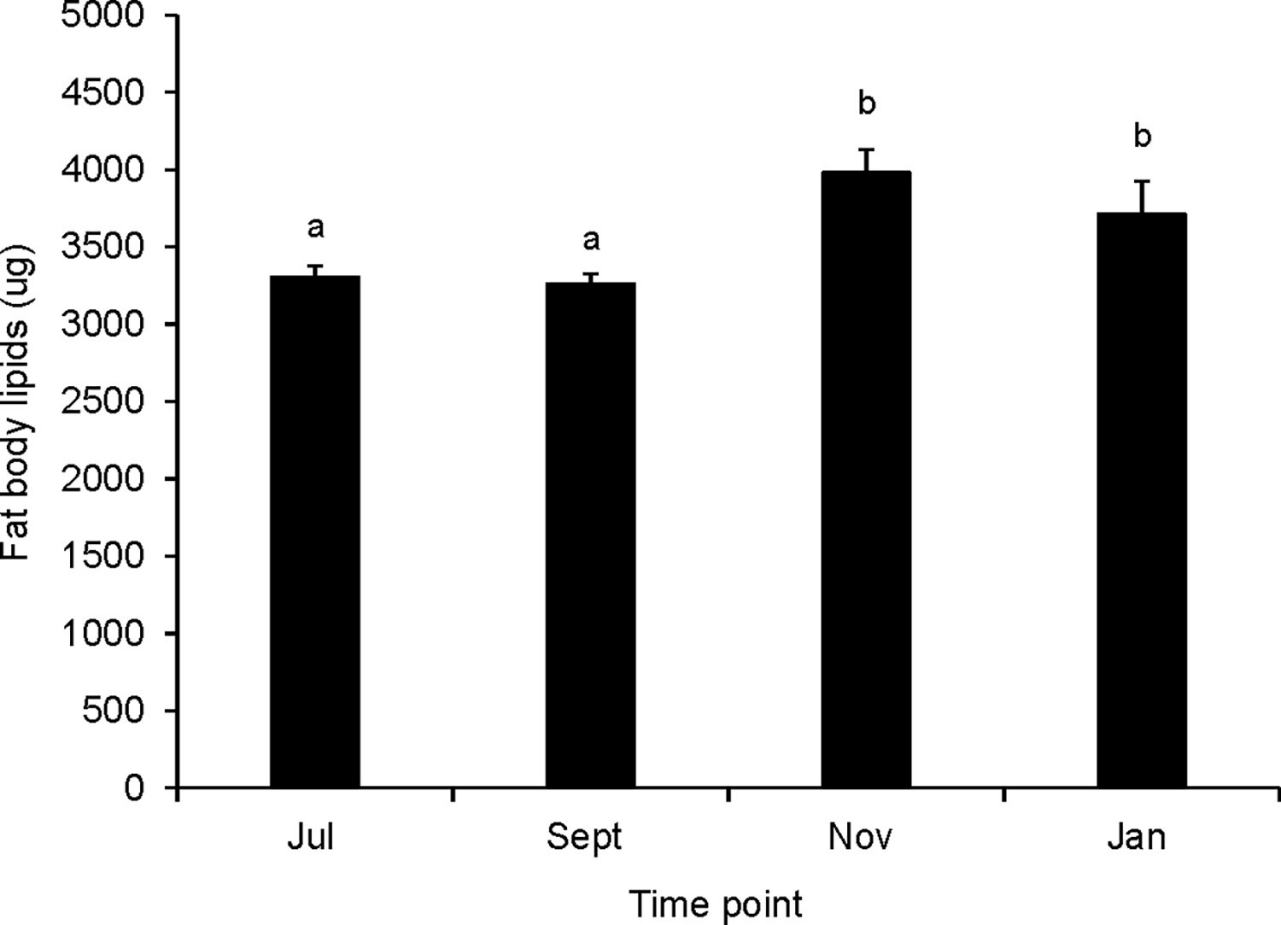
Total lipid contents of fat bodies from queens sampled at different seasonal time points. Error bars represent the standard error (N = 15 to 39 queens at each time point; groups that do not share the same superscript differ by DSCF multiple comparisons (queen ranks; p<0.05)).

Queens also varied in the amount of nutrients stored in non-reproductive tissues, namely their fat bodies. Queens sampled in September and November (but not January) had significantly less soluble protein present in their fat bodies than July queens (Fig 5, one-way ANOVA, F = 4.41, df = 3, p = 0.006). Curiously, fall and mid-winter queens that had lower ovary lipid stores appeared to have higher stores elsewhere in their bodies. November and January queens stored more lipid in their fat bodies than July or September queens (Fig 6, Kruskal-Wallis test, Χ^2^ = 13.222, df = 3, p = 0.004).

### Physiological age (protein carbonyl content of fat bodies) of queens

Queens marginally did not vary significantly in physiological age as measured by fat body protein carbonyl content (S6 Fig, one-way ANOVA, F = 2.57, df = 3, p = 0.059). The absence of protein carbonyl content differences in time point comparisons suggests that some queens may have been replaced by workers after scheduled beekeeper requeenings of the colonies.

### Queen reproductive quality and QMP residues

Nest worker body residues of individual QMP components did not vary with three key metrics of queen reproductive quality. Residues of four individual QMP compounds (HOB, HVA, 9-HDA, and 9-ODA) were not significantly correlated with three queen reproductive quality characteristics (spermatheca sperm counts, soluble protein contents of ovaries, and lipid contents of ovaries; S7A–S7D, S8A–S8D, and S9A–S9D Figs; (sperm) Spearman correlation coefficient, ρ = -0.05165 to 0.04027, p>0.05; (ovary protein) Pearson correlation coefficient, r = -0.07691 to 0.12086, p>0.05; (ovary lipid) Spearman correlation coefficient, ρ =

-0.15461 to 0.26491, p>0.05).

### QMP residues and worker retinue size

Worker responses to queens (as measured by retinue size) did not vary with differences in QMP residues or queen reproductive quality. The number of workers present in worker retinue formations did not significantly correlate either with any of the four individual QMP compound residues (HOB, HVA, 9-HDA, and 9-ODA; S10A–S10D Fig, Pearson correlation coefficient, r = -0.11054 to 0.08168, p>0.05).

## Discussion

Honey bee colonies are broadly adaptive to a wide variety of climatic conditions and are noted for opportunistic use of ephemeral food resources under changing conditions. Brood rearing in warm subtropical locations often hinges on food availability with reduced brood or broodless periods during extended dearth periods when pollen resources are scarce [55–57,59]. Workers in our experimental colonies supported queens with comparable levels of retinue care and trophallactic feeding from summer through late winter. These similarities in queen care across seasons reflect adequate colony nutrition and continuous brood rearing activities in both source (Imperial Valley) and surrogate (Tucson) colonies. As the sole source of queen nutrition, retinue workers engage in trophallactic behaviors that directly support queen oviposition activities [11,21,75]. Queen care and feeding and queen oviposition occur at some level even when workers themselves are malnourished and brood rearing is reduced. Winter brood rearing activities in warm subtropical locations are often marginal and dependent on colony environmental factors such as colony nutrition, site altitude, and available forage. Honey bees from the Lower Mohave desert (Imperial Valley, CA) and the Lower Sonoran desert (Tucson, AZ) may or may not reduce winter brood rearing activities depending on colony nutrition [59,76]. Recent studies have shown that inclusion of supplemental pollen or pollen forage increases brood rearing in overwintering warm subtropical colonies [55,56,77]. In our study, all colonies in both locations had access to either supplemental pollen or pollen forage in all months and brood rearing occurred continuously, albeit at reduced rates during winter.

These similarities in seasonal queen care and queen quality stand in marked contrast to overwintering declines in temperate colonies that experience a more pronounced winter characterized by absolute forage deprivation, broodless periods, reduced queen feeding, and reduced queen grooming [58, see 57 for review]. Colonies in both the Imperial Valley and Tucson sites experienced climatic conditions that were at least marginally suitable for brood rearing from summer to late winter. Here, factors such as forage availability, supplemental feeding and colony nutrition offset other environmental cues that promote entry into broodless overwintering states. In temperate colonies, cues that trigger colony entry into dearth states tend to be cumulative or sustained. Noted induction factors including reduced photoperiod, forage cessation, stored pollen depletion, and reduced temperatures fundamentally contribute to adaptive changes into a broodless overwintering state [54,57]. These factors are less extreme and often more variable year-to-year in warm subtropical locations, with colonies only completely ceasing brood rearing for extended periods under unfavorable conditions.

Notably, workers maintained well-mated queens with nutrient rich, mature ovaries and stored sperm regardless of season or queen age (time since beekeeper requeening). Queens did not vary significantly in key reproductive characteristics acquired during early adult queen maturation (i.e. ovariole number and spermatheca sperm counts) although spermatheca sperm counts were moderately low among many queens [43,47,78–80]. These subtropical queens also did not experience seasonal reduction of ovaries to the atrophied state observed in overwintering Canadian bees by Shehata and coworkers [58]. Instead, queens showed seasonal variation in nutrient resources used to directly support oviposition activity (ovary soluble protein and total lipid contents). Queens in more active brood rearing periods (July and September) generally had more ovary nutrient contents than queens from less active brood rearing periods (January). By contrast, late fall and mid-winter queens appeared to store relatively more nutrients in non-reproductive tissues such as the fat bodies. Curiously, our queens showed similar, although much less pronounced, seasonal ovary growth patterns as queens overwintering in broodless colonies in temperate Canada [58]. Similar seasonal trends have also been observed for ovary development in overwintering worker honey bees [81]. The increase in non-reproductive nutrient stores in mid-winter queens may allow provisioning of queen nutrient stores before the late winter/early spring brood expansion in both climates [58]. Maintenance of active ovaries also may allow subtropical bees to hedge on rapid resumption of brood rearing if sufficient winter ephemeral forage appears [59,71]. Subtropical workers also partially engage in adaptive behaviors and physiological states observed in temperate overwintering colonies. Overwintering colonies from both subtropical locations produce a subpopulation of long-lived, dearth resistant diutinus workers (“winter bees”) and form clusters around the nest center during cold periods [59,76].

Honey bee workers maintain viable queens across a wide array of colony stressors despite seasonal variation in the queen’s signaling. The honest signal theory predicts that workers detect and respond to differences in queen reproductive quality projected by the queen through her queen pheromones [51]. Queen pheromone signaling allows workers to decouple perceptions of queen quality from oviposition activity and brood production and assess queen suitability during both brood rearing and broodless periods [9]. However, our results suggest that QMP transfers varied seasonally, but did not closely reflect differences in queen reproductive quality. Furthermore, retinue workers appeared to tolerate or compensate for broad seasonal differences in QMP profiles. In our experiment, queens of relatively similar quality transferred different amounts of QMP compounds through the year, yet workers provided comparable levels of retinue care across time points. The number of workers observed in our retinues approached the upper limit previously reported in other colony observation studies [21,47]. Part of the lack of differentiation in queen care may have been the relatively high queen quality of these well-mated queens [43,47]. Alternatively, the surrogate colonies may have had high QMP responding workers that responded strongly to queens regardless of QMP differences [45,82]. Most previous studies that noted differences in retinue worker responses to QMP profiles compared queens or queen extracts that differed much more fundamentally in reproductive quality, such as virgin queens and well-mated queens [25,39–42,46,48].

Much to our surprise, queens from the Imperial Valley colonies were remarkably similar in matedness and physiological age across queen age groups despite large differences in time elapsed since beekeeper requeening [59,69,70]. Substandard queens (i.e. unmated, severely sperm-depleted, underdeveloped ovaries, half-caste, injured or diseased queens) were almost completely absent from the sampled colonies. Such similarities suggest that colonies successfully maintained queens of a certain quality and that poorly-mated or severely sperm-depleted queens were either culled or resulted in colony failure. However, annual prophylactic requeening practices by beekeepers may avoid the substantial colony production costs and risks associated with natural queen supersedure [17,54,83]. Honey bee workers behave differently toward unmated/poorly-mated queens and well-mated queens in live queen bioassays [26,42,43,47,50]. Unmated queens have distinctly different QMP compound profiles than well-mated queens that are characterized by much lower or non-existent HVA levels [26]. Conspicuously, we observed a sharp increase in mid-winter HVA residues, a characteristic that has been noted in other well-mated mid-winter queens in warm subtropical colonies [56]. This QMP component distinguishes well mated queens from poorly mated or unmated queens and may serve as an emphatic signal of the queen’s fecundity at a time when queen replacement source material (young worker larvae) first becomes widely available [26]. One factor that should also be considered is that queens that were not superseded aged considerably over the six month longitudinal experiment. Commercial queens last a median time of 11 to 26 months before supersedure or loss [16,84]. Queen QMP composition changes and queen acceptance (survival after introduction) improves with matedness and age among young adult queens [85,86]. Workers likely compensate for differences that arise with ageing among older established mated queens to allow viable queens to be supported.

Most critically, workers appeared to tolerate late fall and winter QMP exposures that were different than summer QMP levels. Despite large differences in QMP residues and near continuous access to young larvae, our field observations noted that workers from both source and receiving colonies rarely produced fully formed replacement queen cells. The costs of queen replacement is severe for productive colonies in terms of lost worker productivity and the risk of new queen loss [54,83]. Furthermore, mature drones needed for queen mating are much less available during extended dearth periods, if at all [11]. Overwintering workers that react to changes in QMP profiles by supersedure risk colony loss through displacement of a mated queen with a terminal virgin queen. The similarities in retinue responses across QMP residue differences suggests, too, that other factors may contribute to retinue interactions. Previous studies on queens with surgically-removed mandibular glands have demonstrated that these queens are capable of attracting retinue workers without QMP pheromones [37]. Honey bee queens produce other pheromones and chemical cues such as tergal gland emissions, β-ocimene, Dufour’s gland emissions, and cuticular hydrocarbons whose full effects on workers are not fully understood [34,35,65,66].

Seasonal and stress-related variation in emissions has been observed with other colony pheromones, both in terms of individual pheromone production and mass effects based on producer subpopulation size. Brood-derived primer pheromones β-ocimene and Brood Ester Pheromone (BEP) are produced by young larvae/newly capped brood and older larvae, so colony-level concentrations of pheromones are a function of their subpopulation sizes [87–89]. Individual adult worker production of the primer pheromone ethyl oleate, which slows adult worker maturation into foragers, is seasonally lower in overwintering bees [90]. Honey bee pheromone production also varies with the state of individual producers. Starving young larvae produce more β-ocimene, which increases worker feeding of brood, than well fed young larvae [91]. Queen pheromones differ from these colony pheromones in that only one individual normally produces significant amounts of these cues. However, the worker base that supports the queen varies considerably in quality and task inclinations through the year.

It remains to be determined how and why workers tolerate such variable shifts in QMP signaling without notable changes in their responses. Our understanding of how QMP and other pheromone signaling pathways vary under colony stressors is largely limited to primary mechanisms of pheromone production and development, yet understudied in other key aspects [9,37]. For example, effective exposure rates of individual workers to colony pheromones may seasonally vary with changes in population size and age structure–factors that directly affect the number of producers and receivers involved in these exchanges [92]. Overwintering colonies are often smaller than their summer counterparts and may not require the same amounts of pheromones to achieve similar receiver exposures. Even less understood are seasonal differences in pheromone receiver perception and responses (see [45,82,93] for variation in individual worker responses). Diutinus workers produced during severe winters and other extended dearths differ from summer workers not only in terms of longevity and physiology, but also in behavioral attributes and nursing capabilities [94,95]. Diutinus workers may be more tolerant of variable QMP profiles to compensate for more unpredictable colony outcomes experienced during dearth periods. Untimely queen events such as late summer requeenings have been shown to disrupt the production of diutinus workers in temperate colonies [54]. Recent studies on overwintering worker longevity in Tucson colonies indicates that only part of the worker population shows extended lifespans associated with the diutinus states. Subtropical colonies may maintain a mixture of diutinus and “summer” workers to provide flexibility when dearth conditions are more marginal and less predictable. Honey bees show many individual and collective adaptations to poor nutrition, overwintering, and other significant colony stressors. Future work will address how bees reinterpret the cues and communication systems that collectively maximize future colony production in marginal times.

## Supporting information

S1 Fig Successful and attempted queen feeding bouts by retinue workers at different seasonal time points. Both successful and attempted feedings of workers enclosed with each queen in the 30 minute observation period were enumerated. Error bars represent the standard error (N = 16 to 39 queens at each time point).
(TIF)

S2 Fig PCA analysis of QMP residues recovered from nest worker bodies at different seasonal time points. Four components (methyl-*p-*hydroxybenzoate (HOB), 4-hydroxy-3-methoxyphenylethanol (HVA), 9-hydroxy-2-decenoic acid (9-HDA) and 9-oxo-2-decenoic acid (9-ODA)) were collected from retinue worker bodies enclosed with the queen. The two enantiomers of 9-HDA are reported together since these were largely indistiguishable. PC1 and PC2 explain 42.5% and 25.3% of the variance respectively. Proportions of these four QMP compound residues differed between seasonal time point groups as indicated by comparisons across each principal component (Kruskal-Wallis test, Χ^2^ = 49.445, df = 3, p<0.0001 (PC1); Χ^2^ = 37.098, df = 3, p<0.0001 (PC2)). Color-coded ellipses indicate 95% confidence levels for PC1 and PC2 on the scatterplot for each indicated treatment group.
(TIF)

S3 Fig Queen fresh body mass at different seasonal time points. Error bars represent the standard error (N = 16 to 39 queens at each time point; none of the means differed by Tukey’s post hoc test (queen means; p>0.05)).
(TIF)

S4 Fig Sperm contents of spermathecae from queens sampled at different seasonal time points. Error bars represent the standard error (N = 16 to 39 queens at each time point; none of the ranks differed by DSCF multiple comparisons).
(TIF)

S5 Fig Average ovariole number of the right ovary of queens sampled at different seasonal time points. Error bars represent the standard error (N = 16 to 39 queens at each time point; none of the means differed by Tukey’s post hoc test (queen means; p>0.05)).
(TIF)

S6 Fig Fat body protein carbonyl contents (physiological age proxy) of queens from different seasonal time points. Error bars represent the standard error (N = 16 to 39 queens at each time point; none of the means differed by Tukey’s post hoc test (queen means; p>0.05)).
(TIF)

S7 Fig a-d. Correlations between queen spermatheca sperm counts and QMP residues at different seasonal time points. Four QMP compounds a) 9-HDA (both enantiomers), b) 9-ODA, c) HOB, and d) HVA were characterized from workers enclosed with each queen (N = 16 to 39 queens at each time point). The Spearman correlation coefficient represents all queens at all time points (p>0.05 after Bonferroni corrections).
(TIF)

S8 Fig a-d. Correlations between queen ovary protein contents of and QMP residues at different seasonal time points. Four QMP compounds a) 9-HDA (both enantiomers), b) 9-ODA, c) HOB, and d) HVA were characterized from workers enclosed with each queen (N = 16 to 39 queens at each time point). The Pearson correlation coefficient represents all queens at all time points (p>0.05 after Bonferroni corrections).
(TIF)

S9 Fig a-d. Correlations between queen ovary lipid contents and QMP residues at different seasonal time points. Four QMP compounds a) 9-HDA (both enantiomers), b) 9-ODA, c) HOB, and d) HVA were characterized from workers enclosed with each queen (N = 16 to 39 queens at each time point). The Spearman correlation coefficient represents all queens at all time points (p>0.05 after Bonferroni corrections).
(TIF)

S10 Fig a-d. Correlations between queen QMP residues and retinue size at different seasonal time points. Four QMP compounds a) 9-HDA (both enantiomers), b) 9-ODA, c) HOB, and d) HVA were characterized from workers enclosed with each queen (N = 16 to 39 queens at each time point). The Pearson correlation coefficient represents all queens at all time points (p>0.05 after Bonferroni corrections).
(TIF)

S1 File (XLSX)

S2 File (PDF)
