# Supplementary figures and images for: Honey bee retinue workers respond similarly to queens despite seasonal differences in Queen Mandibular Pheromone (QMP) signaling
Source: PLoS One. 2023 Sep 28;18(9):e0291710. doi: 10.1371/journal.pone.0291710 (PMC10538780; doi:10.1371/journal.pone.0291710)

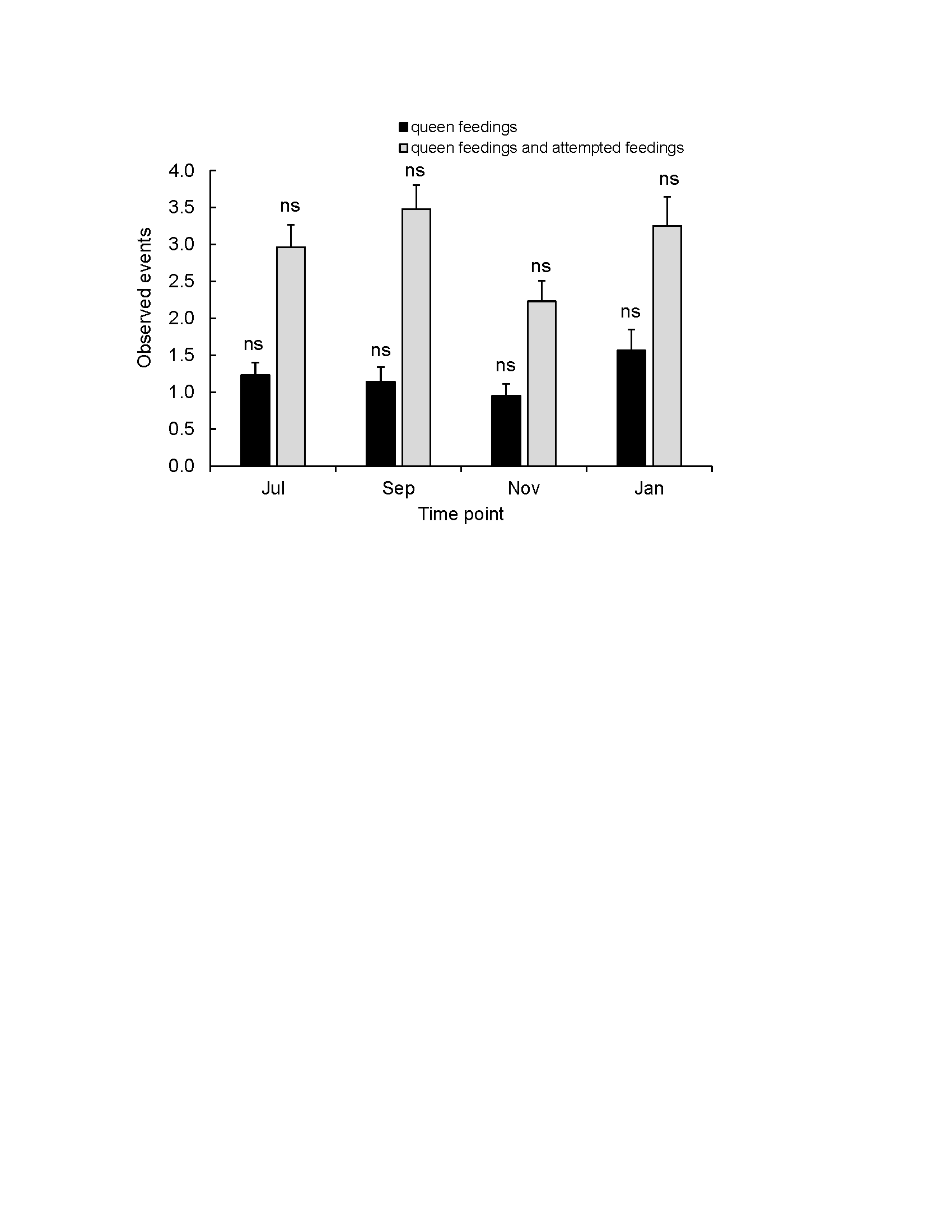

Supplement: S1 Fig — Both successful and attempted feedings of workers enclosed with each queen in the 30 minute observation period were enumerated. Error bars represent the standard error (N = 16 to 39 queens at each time point). (TIF) [file pone.0291710.s001.tif]

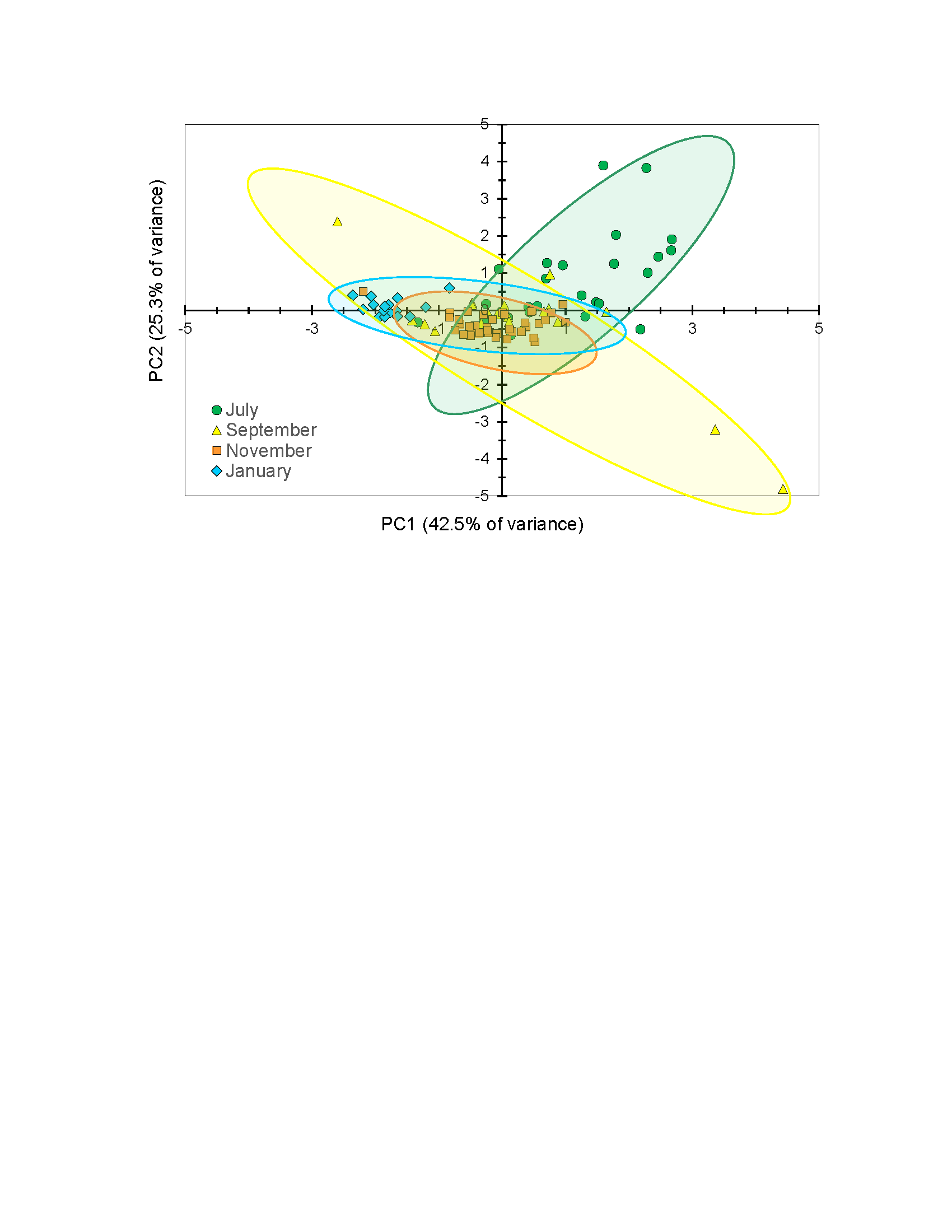

Supplement: S2 Fig — Four components (methyl-p-hydroxybenzoate (HOB), 4-hydroxy-3-methoxyphenylethanol (HVA), 9-hydroxy-2-decenoic acid (9-HDA) and 9-oxo-2-decenoic acid (9-ODA)) were collected from retinue worker bodies enclosed with the queen. The two enantiomers of 9-HDA are reported together since these were largely indistiguishable. PC1 and PC2 explain 42.5% and 25.3% of the variance respectively. Proportions of these four QMP compound residues differed between seasonal time point groups as indicated by comparisons across each principal component (Kruskal-Wallis test, Χ2 = 49.445, df = 3, p<0.0001 (PC1); Χ2 = 37.098, df = 3, p<0.0001 (PC2)). Color-coded ellipses indicate 95% confidence levels for PC1 and PC2 on the scatterplot for each indicated treatment group. (TIF) [file pone.0291710.s002.tif]

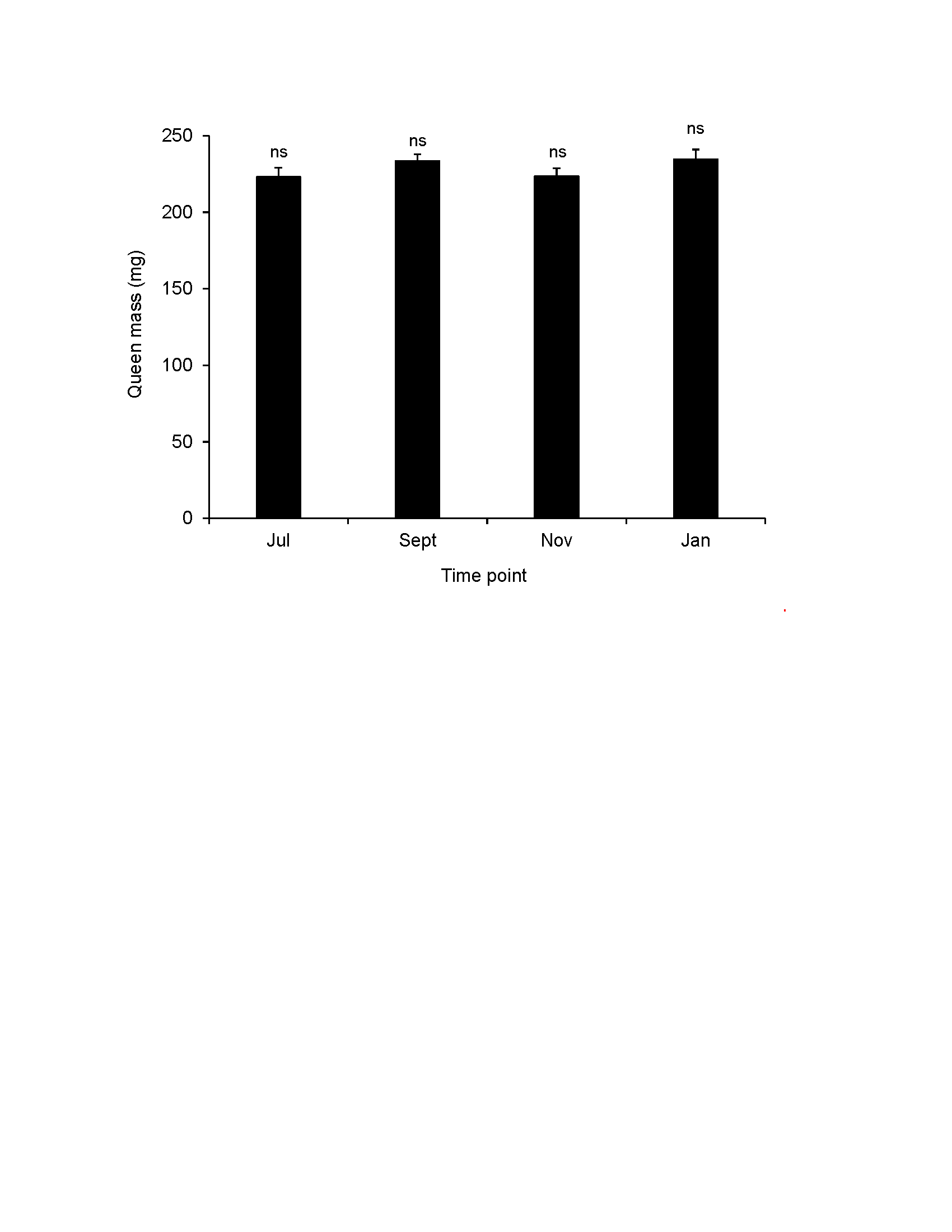

Supplement: S3 Fig — Error bars represent the standard error (N = 16 to 39 queens at each time point; none of the means differed by Tukey’s post hoc test (queen means; p>0.05)). (TIF) [file pone.0291710.s003.tif]

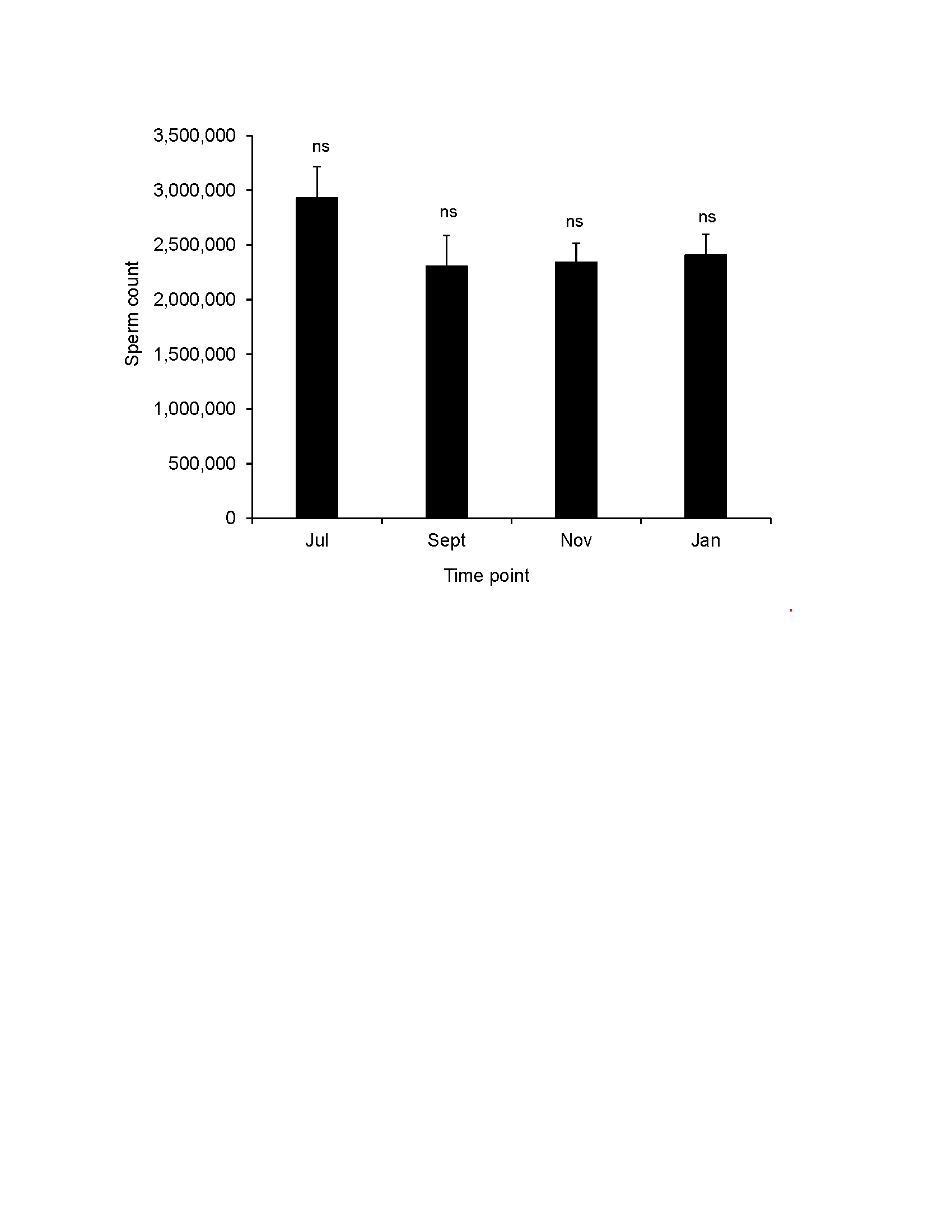

Supplement: S4 Fig — Error bars represent the standard error (N = 16 to 39 queens at each time point; none of the ranks differed by DSCF multiple comparisons). (TIF) [file pone.0291710.s004.tif]

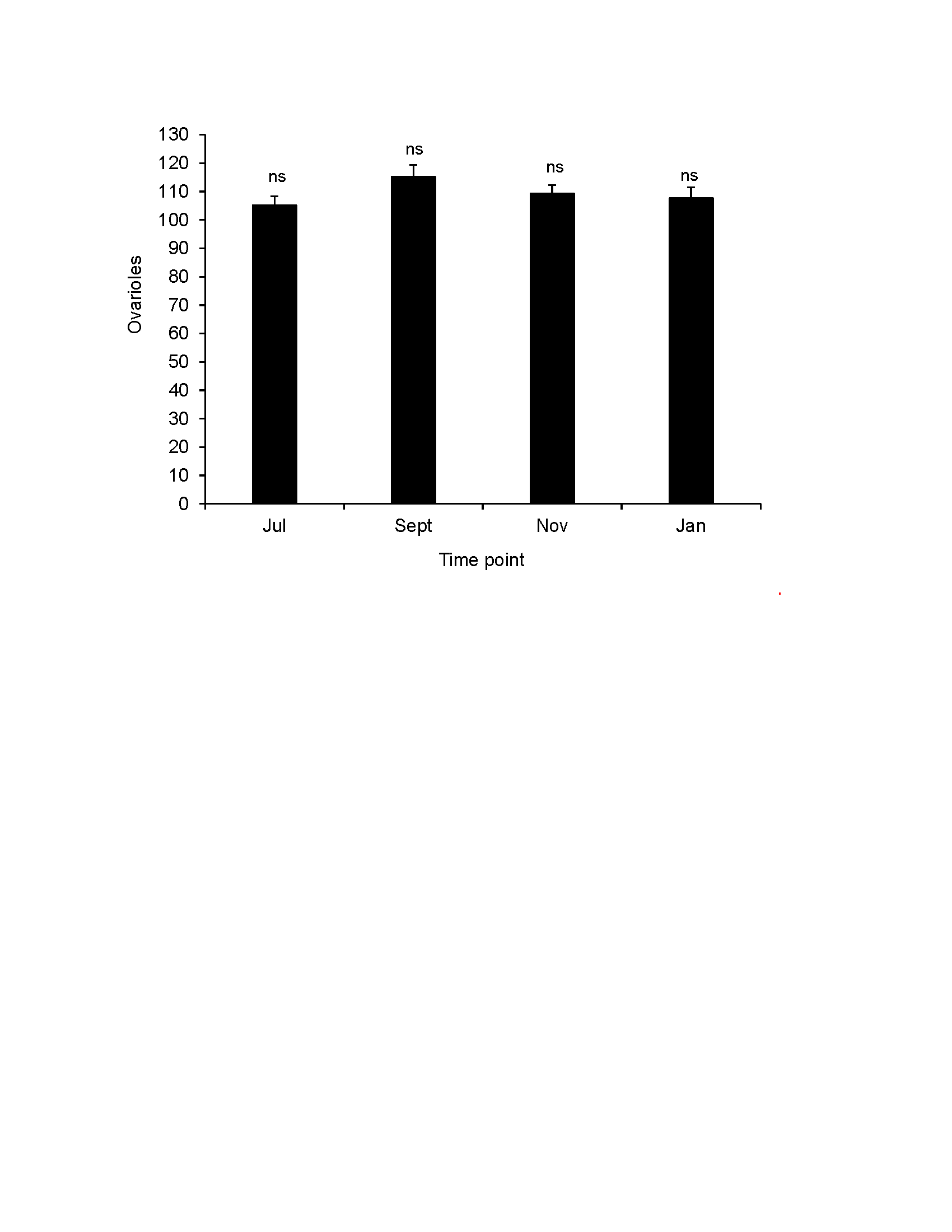

Supplement: S5 Fig — Error bars represent the standard error (N = 16 to 39 queens at each time point; none of the means differed by Tukey’s post hoc test (queen means; p>0.05)). (TIF) [file pone.0291710.s005.tif]

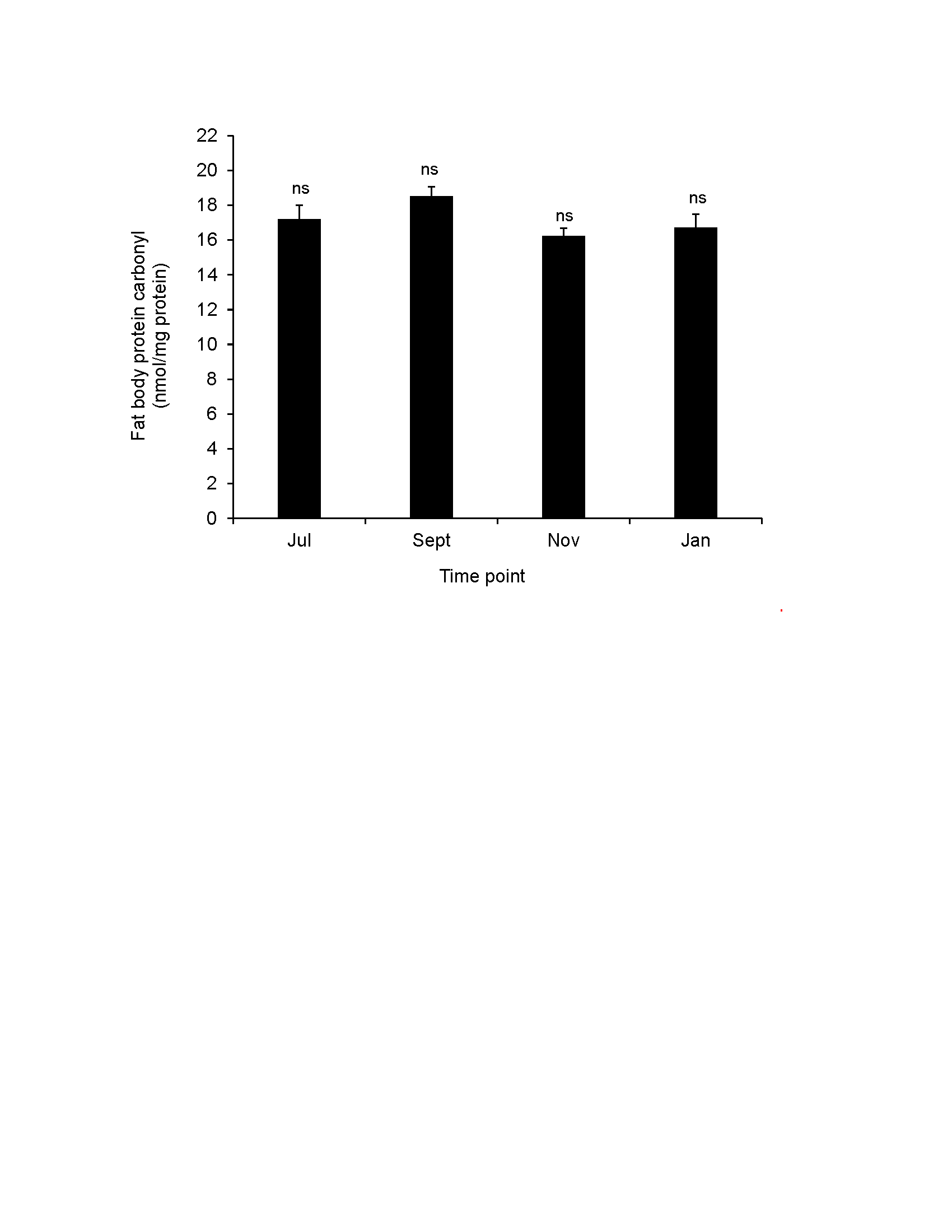

Supplement: S6 Fig — Error bars represent the standard error (N = 16 to 39 queens at each time point; none of the means differed by Tukey’s post hoc test (queen means; p>0.05)). (TIF) [file pone.0291710.s006.tif]

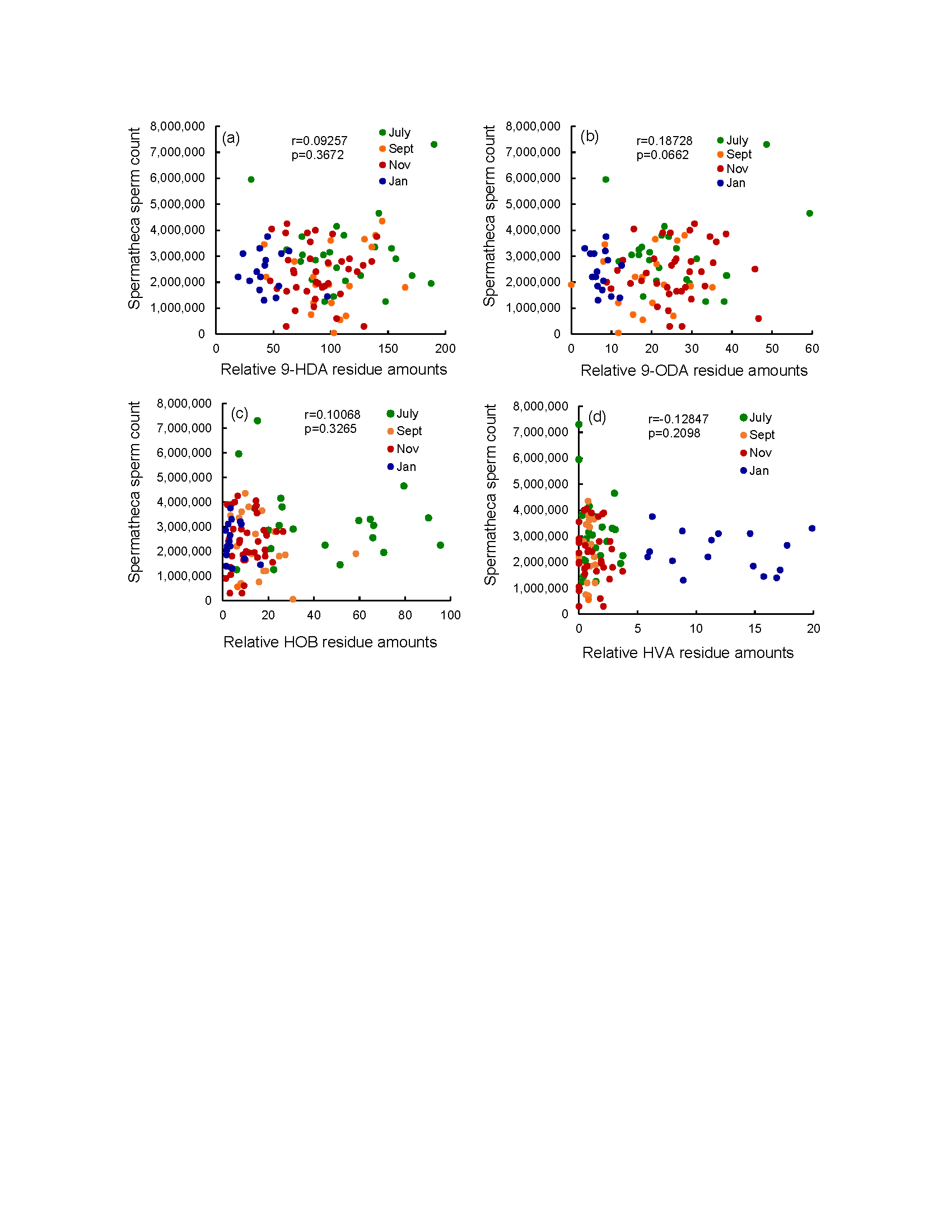

Supplement: S7 Fig — a-d. Correlations between queen spermatheca sperm counts and QMP residues at different seasonal time points. Four QMP compounds a) 9-HDA (both enantiomers), b) 9-ODA, c) HOB, and d) HVA were characterized from workers enclosed with each queen (N = 16 to 39 queens at each time point). The Spearman correlation coefficient represents all queens at all time points (p>0.05 after Bonferroni corrections). (TIF) [file pone.0291710.s007.tif]

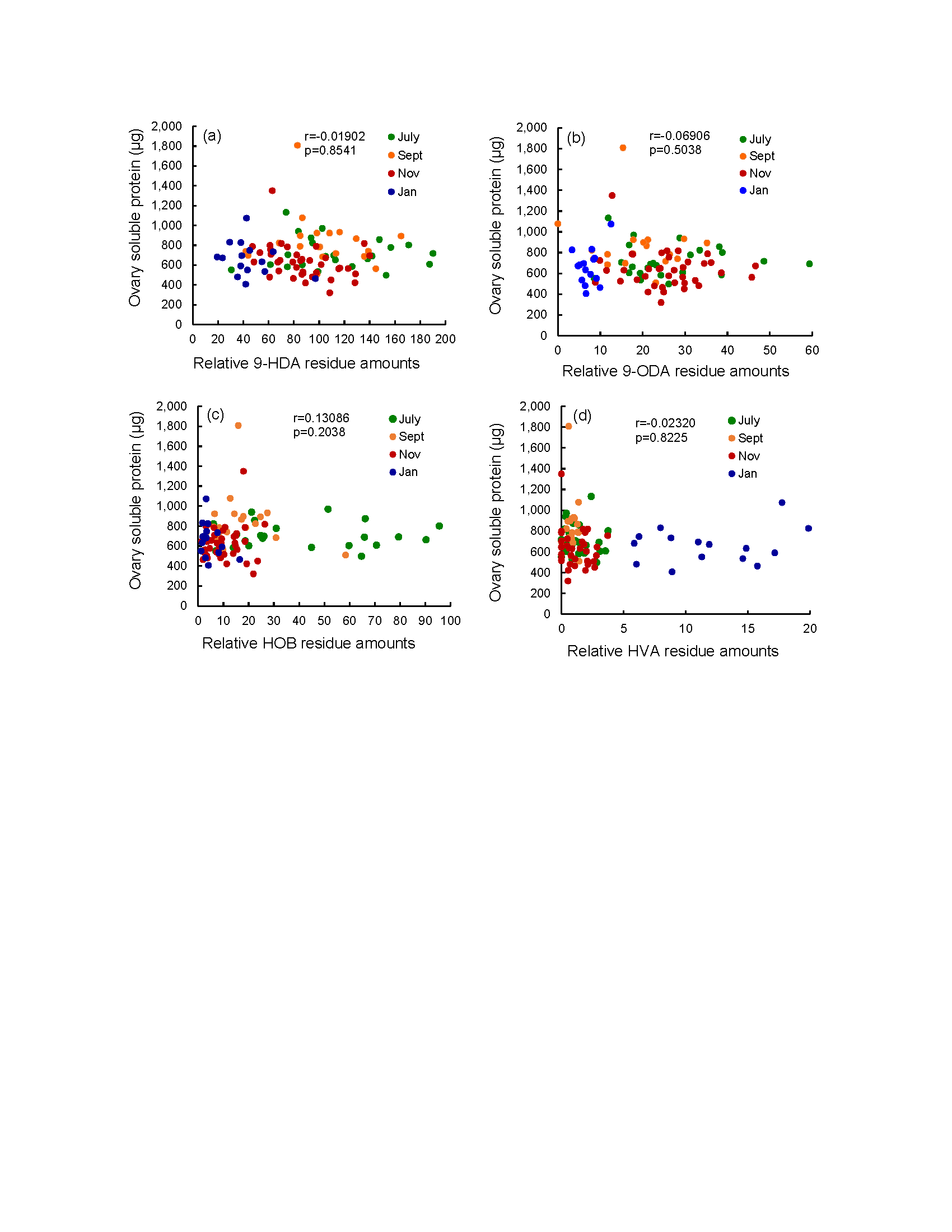

Supplement: S8 Fig — a-d. Correlations between queen ovary protein contents of and QMP residues at different seasonal time points. Four QMP compounds a) 9-HDA (both enantiomers), b) 9-ODA, c) HOB, and d) HVA were characterized from workers enclosed with each queen (N = 16 to 39 queens at each time point). The Pearson correlation coefficient represents all queens at all time points (p>0.05 after Bonferroni corrections). (TIF) [file pone.0291710.s008.tif]

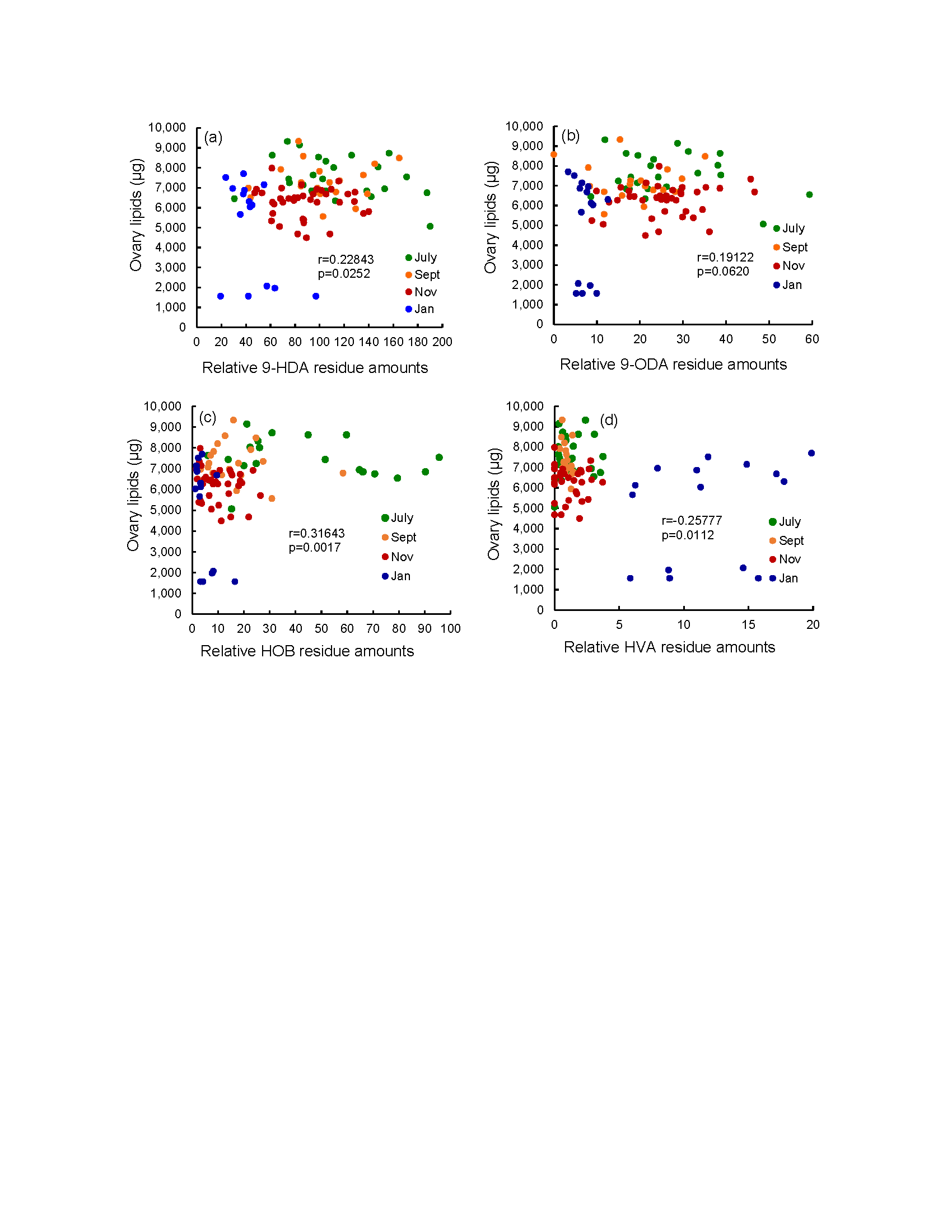

Supplement: S9 Fig — a-d. Correlations between queen ovary lipid contents and QMP residues at different seasonal time points. Four QMP compounds a) 9-HDA (both enantiomers), b) 9-ODA, c) HOB, and d) HVA were characterized from workers enclosed with each queen (N = 16 to 39 queens at each time point). The Spearman correlation coefficient represents all queens at all time points (p>0.05 after Bonferroni corrections). (TIF) [file pone.0291710.s009.tif]

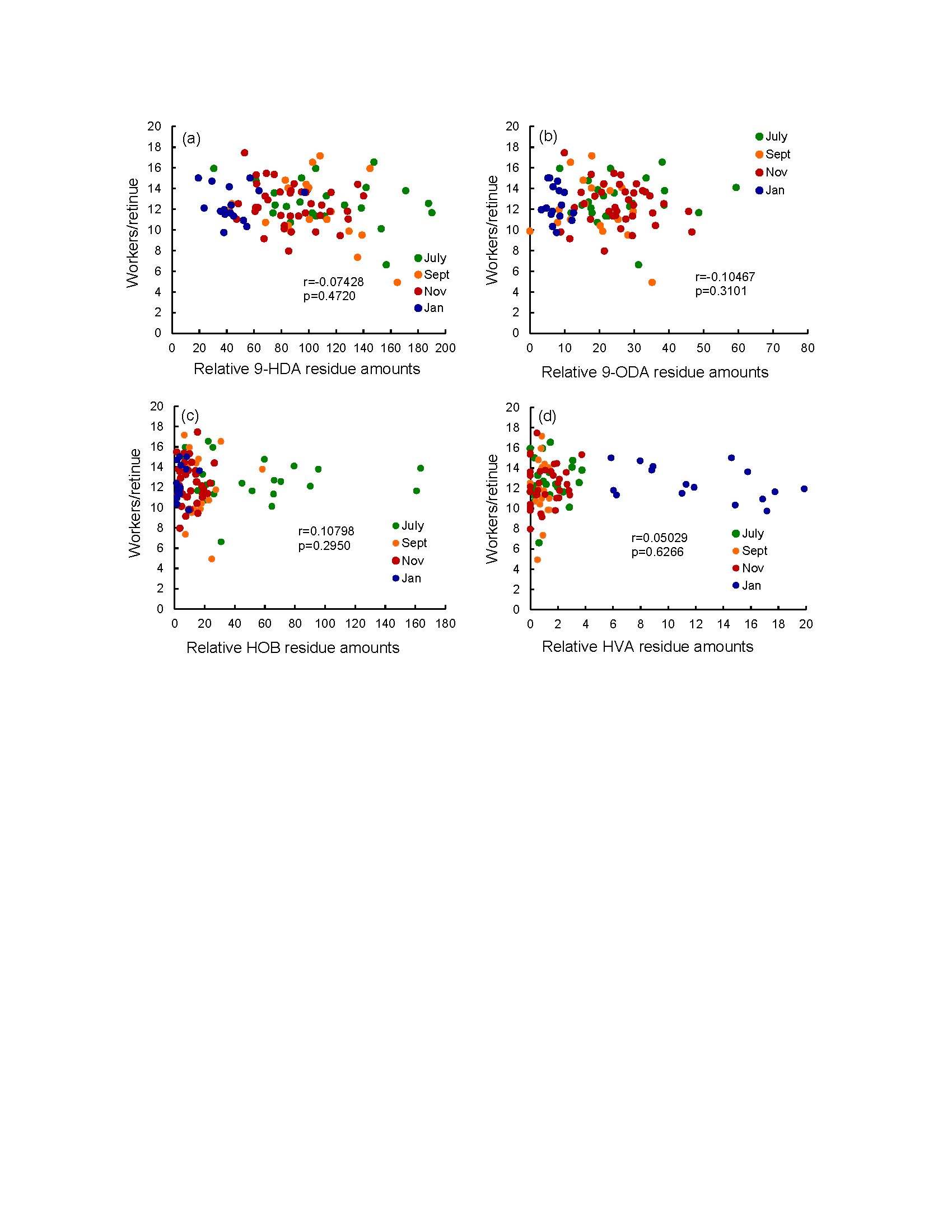

Supplement: S10 Fig — a-d. Correlations between queen QMP residues and retinue size at different seasonal time points. Four QMP compounds a) 9-HDA (both enantiomers), b) 9-ODA, c) HOB, and d) HVA were characterized from workers enclosed with each queen (N = 16 to 39 queens at each time point). The Pearson correlation coefficient represents all queens at all time points (p>0.05 after Bonferroni corrections). (TIF) [file pone.0291710.s010.tif]
