# Supplementary material for: Honey bee retinue workers respond similarly to queens despite seasonal differences in Queen Mandibular Pheromone (QMP) signaling
Source: PLoS One. 2023 Sep 28;18(9):e0291710. doi: 10.1371/journal.pone.0291710 (PMC10538780; doi:10.1371/journal.pone.0291710)
Supplement: S2 File — (PDF) [file pone.0291710.s012.pdf]

S12_File

Annotation for S11_File data sets for PLOS ONE "Honey bee retinue workers respond similarly to queens despite seasonal differences in Queen Mandibular Pheromone (QMP) signaling"

Physiological metrics

Queen code is the sample code for the queen.

Queen colony code refers to the Imperial Valley colony coding and is a secondary identifier.

Time point refers to the seasonal time point that the queen was removed from a source Imperial Valley colony to a surrogate Tucson colony.

Queen body wet mass is the mass of the frozen queen (taken after she was sampled at the end of the retinue observations).

Spermatheca sperm counts were estimated from a hemocytometer preparation of a spermatheca sperm suspension (as detailed in the methods). Sperm were counted on five etched hemocytometer squares at random and averaged. The average sperm count was multiplied by 250,000 to yield an estimate of the total number of sperm stored in the spermatheca.

Right ovariole counts were made by sectioning the right ovariole under a dissecting microscope and carefully teasing apart the ovarioles.

Total soluble protein content was estimated from ovaries and fat bodies homogenates using a Pierce BCA protein assay kit (Thermo Fisher Scientific, Waltham, MA, USA) bicinchoninic acid assay. Samples were analyzed by kit instructions. Different amounts of tissue homogenate subsamples were analyzed in order to match the linear range of the standard curve.

Total lipid content was estimated from ovaries and fat bodies homogenates using a modified chromic acid assay [72]. Different amounts of tissue homogenate subsamples were analyzed in order to match the linear range of the standard curve.

Ovaries homogenate preparation. Both ovaries were homogenized for 30 sec by Bead Beater in 2.000 mL 100 mM pH 7.4 HEPES buffer and briefly centrifuged to pellet solids.

Soluble protein contents of ovaries. A 25 µL subsample was combined with 175 µL BCA reactant in a well of a 96 well plate at 32°C and compared against a bovine saline albumin (BSA) standard curve at 562 nm absorbance in a Gen-5 Plate Reader (Biotek, Inc., Winooski, UT, USA). Samples were run in triplicate to obtain an average net 562 nm absorbance and corrected for the subsample fraction taken.

Total lipid contents of ovaries. A Folch extraction of ovaries was made by combining 510 µL ovary homogenate with 1000 µL chloroform: methanol, homogenizing the mixture for 30 sec, and centrifuging the mixture to obtain a partitioned Folch extraction. Exactly 80 µL of the lower chloroform: methanol layer was transferred to a crimp cap vial, dried down in a SpeedVac, and reacted with 1.000 mL chromic acid on an aluminum block hot plate at 95°C for 1h. A subsample of 200 µL sample reactant transferred onto a 96 well plate and compared against a reacted oleic acid standard curve at 620 nm absorbance in a Gen-5 Plate Reader (Biotek, Inc., Winooski, UT, USA). Samples were run in triplicate to obtain an average net 620 nm absorbance and corrected for the subsample fraction taken.

Fat bodies homogenate preparation. The fat body tissues were homogenized for 30 sec by Bead Beater in 600 µL TE buffer and briefly centrifuged to pellet solids.

Soluble protein contents of fat bodies. A 5 µL subsample was combined with 195 µL BCA reactant in a well of a 96 well plate at 32°C and compared against a bovine saline albumin (BSA) standard curve at 562 nm absorbance in a Gen-5 Plate Reader (Biotek, Inc., Winooski, UT, USA). Samples were run in triplicate to obtain an average net 562 nm absorbance and corrected for the subsample fraction taken.

Total lipid contents of fat bodies. A Folch extraction of fat bodies was made by combining 300 µL fat bodies homogenate with 1000 µL chloroform: methanol, homogenizing the mixture for 30 sec, and centrifuging the mixture to obtain a partitioned Folch extraction. Exactly 80 µL of the lower chloroform: methanol layer was transferred to a crimp cap vial, dried down in a SpeedVac, and reacted with 1.000 mL chromic acid on an aluminum block hot plate at 95°C for 1h. A subsample of 200 µL sample reactant transferred onto a 96 well plate and compared against a reacted oleic acid standard curve at 620 nm absorbance in a Gen-5 Plate Reader (Biotek, Inc., Winooski, UT, USA). Samples were run in triplicate to obtain an average net 620 nm absorbance and corrected for the subsample fraction taken.

Protein carbonyl content of fat bodies. The relative physiological age of queens was estimated by quantifying accumulated protein oxidation (protein carbonyl contents) in fat bodies tissues homogenates using a Protein Carbonyl Content Assay kit (Sigma, St. Louis, MO, USA)[60]. A 400 µL fat bodies homogenate supernatant was incubated with 10% streptozocin to precipitate nucleic acids, reacted with 2,4-dinitrophenylhydrazine (DNPH), purified, and processed by the kit protocol to obtain the protein carbonyl product (dinitrophenyl hydrazone adduct). Total soluble protein content was determined by resuspending purified protein pellets in 100 µL 6M guanidine solution, transferring the suspension to a 96 well plate, and reading 375 nm absorbance in a Gen-5 Plate Reader (Biotek, Inc., Winooski, UT, USA). Total protein carbonyl content of the fat body homogenate was calculated by applying the protein carbonyl product (dinitrophenyl hydrazone adduct) extinction coefficient to the sample reading. The protein carbonyl content was scaled against the total soluble protein content and corrected for the subsample fraction taken.

Retinue metrics

Queen code is the sample code for the queen.

Queen colony code refers to the Imperial Valley colony coding and is a secondary identifier.

Time point refers to the seasonal time point that the queen was removed from a source Imperial Valley colony to a surrogate Tucson colony.

Each videorecorded retinue worker-queen observation session is summarized by queen and month in the retinue data tabs. Workers were rated as retinue workers if they were 1) touching the queen, 2) oriented toward the queen, or 3) stationary or nearly stationary for 10 seconds facing the queen (criteria after Seeley, 1979 [21]). The retinue counts included workers that were outside the inner circle of retinue workers (ones adjacent to the queen) that fulfilled the latter two criteria. The **top number of retinue workers observed for each minute** is listed with the **top three retinue counts averaged**. This **average of the top three retinues** is included in the retinue metrics tab and is the basis for comparison.

Number of workers that successfully fed the queen refer to the number of workers observed matching all three of van der Blom 1992 [22] criteria (1) the worker remaining stationary facing the queen, 2) the extension of worker mouthparts toward the queen, and 3) the extension of queen mouthparts to engage in trophallactic feeding). **Number of workers that attempted to feed the queen but were rejected** refers to the number of workers that only met the first two criteria but were not reciprocated by the queen. **Total number of workers that fed and attempted to feed the queen** is the sum of these two groups.

QMP residues

QMP residues were calculated as the relative compound amounts present in the sample. Relative amounts were compared rather than absolute amounts due to concerns about the stability of the 9-ODA and 9-HDA standards in long term cold storage (some degradation may have occurred despite storage at -80°C). Relative compound amounts were calculated as:

(peak area/compound peak sensitivity)*correction factor for fraction injected

The correction factor for the fraction of the sample injected was calculated from the peak area of the internal standard (cis-10-heptadecenoic acid) detected in the sample.

Characteristic ions (m/z) used in SIM mode to quantify peak areas of samples and verify against standards.

HOB	224 m/z
HVA	312 m/z
ODA	241 m/z
HDA	315 m/z
cis-10-heptadecenoic acid (cis-10)	325 m/z
